# Patterns of emergence and circulation of West Nile Virus in Algeria

**DOI:** 10.1038/s41467-026-74856-6

**Published:** 2026-07-06

**Authors:** Aissam Hachid, Chahrazed Benbetka, Abderrezak Bouamra, Nesrine Bouredjoul, Fayez Ahmed Khardine, Noé Ochida, Rafik Garni, Aldjia Khaldi, Mohamed Seghier, Kelly Charniga, Simon Cauchemez

**Affiliations:** 1 Laboratory of Arboviruses and Emerging Viruses, Institut Pasteur d’Algérie, Algiers, Algeria; 2 Faculty of Pharmacy, University of Health Sciences, Algiers, Algeria; 3 Center for Perinatal Research, Nationwide Children’s Hospital, Columbus, OH USA; 4 Department of Epidemiology, Beni-Messous University Hospital, Algiers, Algeria; 5 Faculty of Medicine, University of Health Sciences, Algiers, Algeria; 6 Mathematical Modelling of Infectious Diseases Unit, Institut Pasteur, Université Paris Cité, INSERM U1332, CNRS UMR 2000, Paris, France; 7 Department of Preventive Medicine and Medical Analysis, Institut Pasteur d’Algérie, Algiers, Algeria

**Keywords:** Viral infection, West nile virus, Viral transmission

## Abstract

In recent years, many countries have experienced increased outbreaks of vector-borne diseases, such as West Nile virus (WNV). WNV is primarily transmitted to humans by the bite of infected *Culex* mosquitoes and has an enzoonotic transmission cycle involving birds. In Algeria, serological evidence of WNV in humans dates back to the 1970s, but circulation patterns are poorly characterized. To address this, we conduct a cross-sectional seroprevalence survey of WNV in humans and fit serocatalytic models to the data. We also analyze data on severe WNV cases. In this study, we report a seroprevalence of 16.7%. We identify age, region, and residence type as risk factors for infection. We also show that patterns of spread in two provinces in the south are consistent with low level endemic risk over decades, while those from three provinces in the north are all consistent with single outbreaks that occurred within the last 10-15 years. An outbreak may have occurred in Oran in 2010 and in Jijel and Tizi Ouzou near the time of data collection in 2017 and 2018, respectively. Investigation of potential drivers of viral establishment, along with continued surveillance of WNV cases, especially in the northern provinces, is warranted.

## Abstract (web summary)

Authors report seroprevalence of 16.7% for West Nile Virus in Algeria. Age, region, and residence type were risk factors for infection. They also found differences in the patterns of viral spread across provinces in the north versus the south.

## Introduction

West Nile virus (WNV), an enveloped, single-stranded RNA virus^1^ belonging to the family *Flaviviridae* (genus *Orthoflavivirus*; species *Orthoflavivirus nilense*)^2,3^ is a neurotropic arbovirus of public health concern. It is primarily transmitted to humans through the bite of infected mosquitoes, predominantly by *Culex* species, such as *Culex pipiens* complex and *Culex perexiguus*^4^. Other mosquito species, including *Aedes albopictus* and *Aedes rusticus*, have been reported to transmit WNV^5^. In rare cases, person-to-person transmission has been documented through blood transfusion, organ transplantation, and from mother to baby during pregnancy, delivery, or breastfeeding^6^. WNV has an enzootic transmission cycle involving migratory or resident birds, which are the natural reservoirs. Most of these birds act as amplifying hosts^4^. Horses, humans, and other mammals are considered dead-end hosts^7^. In humans, WNV infection is often asymptomatic or presents with mild flu-like symptoms. However, in less than 1% of cases, WNV can cause neurological symptoms, including West Nile Neuroinvasive Disease (WNND), which varies from self-limiting meningitis to life-threatening meningoencephalitis or encephalitis (ME/E)^8^. Risk factors for severe forms of WNV include older age, underlying medical conditions, immune suppression^9^, male sex^10^, and exposure via blood transfusion^10^ or organ transplantation^11^. Infection with WNV is thought to confer lifelong immunity^12^. There is currently no approved vaccine^13^ or specific treatment^14^ for WNV disease in humans.

In 1937, WNV was first isolated from a febrile woman in the West Nile district of Uganda^15^. The disease was subsequently reported in several locations throughout Africa and the Middle East, with occasional cases of neuroinvasive disease^16^. Since the 1990s, recurrent outbreaks of WNND have occurred and continue to expand to new geographic regions^17–19^. These WNND epidemics were first reported in the Maghreb countries (Algeria in 1994, Morocco in 1996, and Tunisia in 1997)^20,21^ before spreading to other parts of the world, including Europe, the Mediterranean Basin^20,21^, and the United States^17^. In 2018, European countries such as Italy, Serbia, and Greece experienced their largest recorded outbreaks, with over 2000 cases and 181 deaths^4^. Viral evolution, ecological changes influencing the abundance and distribution of WNV vectors and hosts (e.g., land use and urbanization), and climate change, through increasing temperatures and fluctuating rainfall, may contribute to the spread and establishment of WNV in different parts of the world^4,9,22–24^.

The Maghreb region has reported evidence of substantial circulation of WNV from epidemiological surveillance and serological studies in recent years^25^. In Tunisia, one study carried out in 2007 estimated seroprevalence against WNV as high as 28% in humans^26^. Furthermore, the country experienced two major outbreaks in 2012 and 2018, with a combined total of 182 neuroinvasive cases and 17 deaths^27,28^. In Morocco, one human and 42 horses died during an outbreak of equine encephalitis caused by WNV in 1996^29^. Two other equine epizootics were reported in 2003 and 2010, resulting in the deaths of 5 and 8 horses, respectively^29^. In 2011, a serosurvey study conducted among humans in Rabat and Kenitra found seroprevalence of WNV of 12% and 19%, respectively^30^.

As part of the Maghreb, Algeria has diverse ecological zones ranging from the coastal Mediterranean climate with hot summers and mild winters in the north to the arid Sahara Desert in the south. There are stagnant water sources (oases) in the south, and the country is geographically located along the path of bird migration routes between Africa and Europe^31^. During the second half of the twentieth century, the country experienced a trend of increasing temperatures and declining in precipitation, which expanded the arid areas^32^. These factors likely contributed to the widespread presence of *Culex* mosquitoes throughout the country and may have also influenced changes in bird migration routes^33^. Serological surveys, particularly in the southern districts of Biskra and Illizi, have demonstrated the presence of WNV in humans and animals since the 1970s^34^. In 1994, the first confirmed clinical cases among humans were reported during an outbreak in Timimoun, resulting in 15 confirmed cases and 2 deaths^35^. This outbreak was followed by sporadic reports of cases in the north of the country, namely in Jijel (2012) and Guelma (2013)^34^. Recent serological surveys of humans^36^, equids^37^, and wild birds^38^ have provided further evidence of WNV circulation in this part of the country.

Here, we sought to gain further insight into the epidemiology of WNV in Algeria. Across five provinces with suitable environmental conditions for WNV transmission and/or previous reports of human cases of WNV disease, we conducted a seroprevalence study as well as WNV disease acute case detection in hospitals among patients with suspected viral neuroinvasive disease. We also fitted a suite of serocatalytic models to reconstruct the history of WNV circulation in different parts of Algeria.

## Results

### Characteristics of the study participants

For the WNV seroprevalence study, a total of 1235 leftover serum samples were collected in 2017 and 2018 from the five provinces (Table 1). Information about sociodemographic characteristics was missing for some participants (e.g., 0.2% for age and sex and 0.6% for residence type). The mean age of participants was 29 (range 1–98) years. Over half (57.4%) of the participants were female, and 70% lived in urban areas. More participants were from provinces in the north (832 across Jijel, Tizi Ouzou, and Oran) than from the south (403 in Timimoun and Biskra).

**Table 1:** Univariable analysis of risk factors for West Nile virus infection in Algeria, 2017–2018. *N* = 1235

Variables	Total *N*	Seropositivity, N (%)	(95% CI)	OR	(95% CI)	*P* value
Age (*N* = 1232, 99.8%)						
0–9	244	16 (6.6)	(3.8–10.4)	Ref.		
10–29	444	60 (13.5)	(10.5–17.0)	2.2	(1.3–4.1)	0.006
30–54	379	83 (21.9)	(17.8–26.4)	4.0	(2.3–7.3)	1e-6
55–64	79	19 (24.1)	(15.1–35.0)	4.5	(2.2–9.4)	4e-5
65+	86	27 (31.4)	(21.8–42.3)	6.5	(3.3–13.1)	7e-8
Sex (*N* = 1233, 99.8%)						
Female	709	122 (17.2)	(14.5–20.2)	1.1	(0.8–1.5)	0.58
Male	524	84 (16.0)	(13.0–19.5)	Ref.		
Province of residence (*N* = 1235, 100%)						
Timimoun	118	53 (44.9)	(35.7–54.3)	9.4	(5.6–16.1)	<2e-16
Biskra	285	83 (29.1)	(23.9–34.8)	4.8	(3.0–7.6)	3e-11
Jijel	303	30 (9.9)	(6.8–13.8)	1.3	(0.7–2.2)	0.38
Tizi Ouzou	165	11 (6.7)	(3.4–11.6)	0.8	(0.4–1.7)	0.60
Oran	364	29 (8.0)	(5.4–11.2)	Ref.		
Region (*N* = 1235, 100%)						
South	403	136 (33.7)	(29.1–38.6)	5.5	(4.0–7.7)	2e-16
North	832	70 (8.4)	(6.6–10.5)	Ref.		
Residence type (*N* = 1227, 99.4%)						
Suburban/rural	355	99 (27.9)	(23.3–32.9)	2.8	(2.0–3.8)	9e-11
Urban	872	107 (12.3)	(10.2–14.6)	Ref.		
Risk factors of exposure to WNV infection						
Blood transfusion (*N* = 1077, 87.2%)						
Yes	45	7 (15.6)	(6.5–29.5)	1.0	(0.4–2.1)	0.95
No	1032	164 (15.9)	(13.7–18.3)	Ref.		
Residence in a mosquito-infested area (*N* = 1071, 86.7%)						
Yes	463	101 (21.8)	(18.1–25.9)	2.1	(1.5–3.0)	7e-6
No	608	70 (11.5)	(9.1–14.3)	Ref.		
Residence near a water reservoir (*N* = 1158, 93.8%)						
Yes	397	48 (12.1)	(9.1–15.7)	0.7	(0.5–1.0)	0.057
No	761	124 (16.3)	(13.7–19.1)	Ref.		
Outdoor activity (*N* = 447, 36.2%)						
Yes	44	12 (27.3)	(15.0–42.8)	2.8	(1.3–5.6)	0.006
No	403	48 (11.9)	(8.9–15.5)	Ref.		

Logistic regression was used to test statistical significance for all variables. No adjustments for multiple comparisons were made. We used 2-tailed tests for all models.

*N* participant number, *CI* confidence interval, *OR* odds ratio, *Ref* reference category.

### WNV seroprevalence

**Fig. 1:**
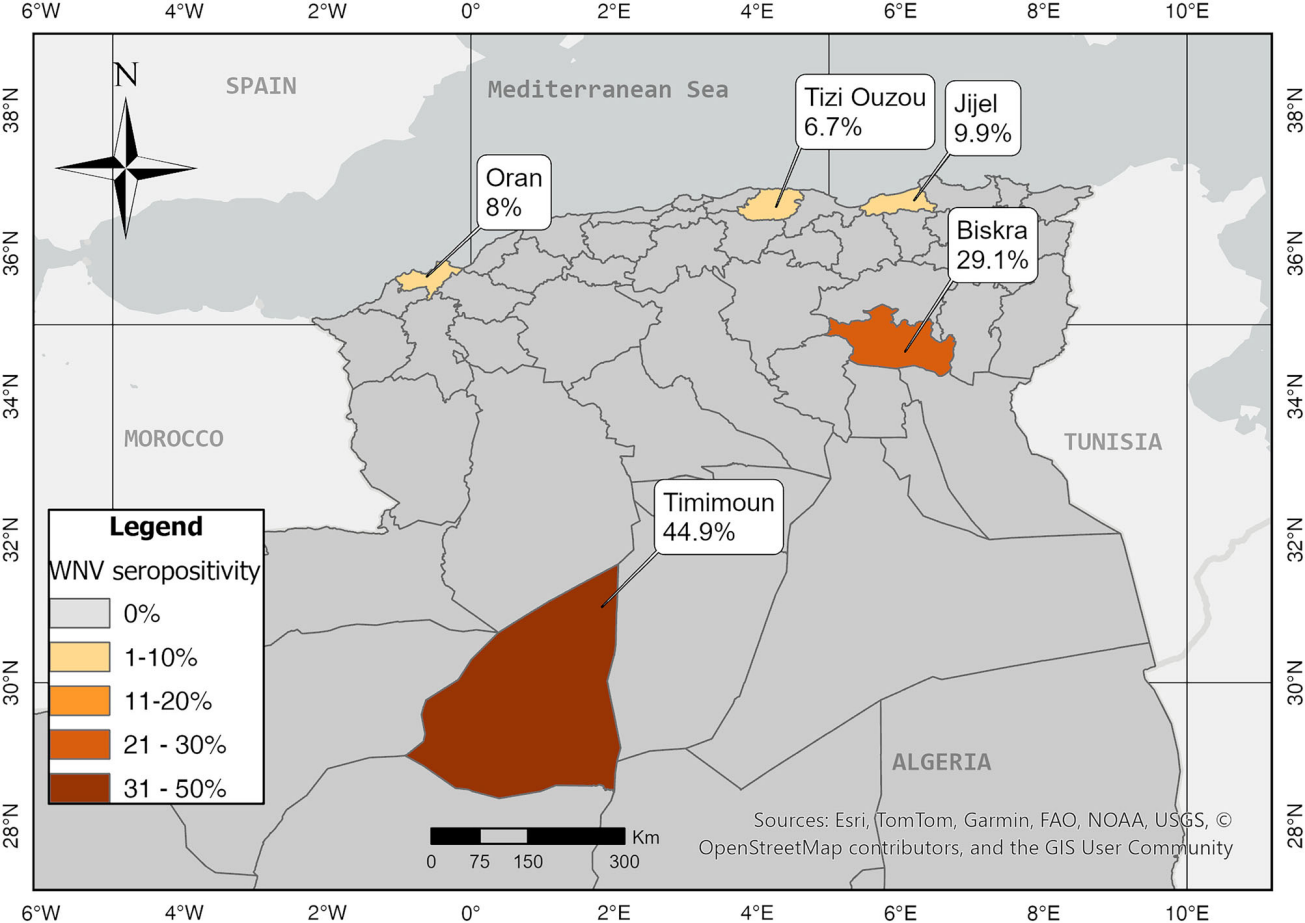
West Nile virus (WNV) seropositivity in Algeria. Map showing the five provinces where the seroprevalence study samples were collected between 2017 and 2018. Colors indicate seropositivity (darker is a higher percentage). Provincial boundaries correspond to administrative divisions prior to the 2019 reorganization.

Out of 1235 samples, 270 were positive or borderline for WNV IgG antibodies by ELISA. Of these 270 individuals, 206 were also positive by PRNT and considered seropositive for WNV. Overall seroprevalence across all study sites was 16.7% (95% CI: 14.6–18.9). WNV seropositivity was higher in areas located in the south than those in the north (Fig. 1).

### Risk factors associated with WNV seropositivity

**Fig. 2:**
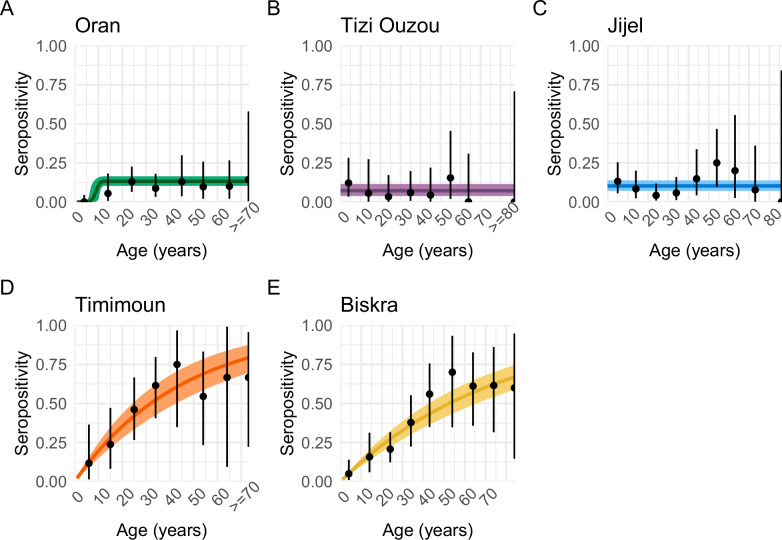
Seropositivity and best-fitting serocatalytic model fits of West Nile virus from a cross-sectional seroprevalence study in Algeria. Sampling for all provinces was performed in 2018, except for Jijel where sampling occurred in 2017. **A** shows model fits with one outbreak in Oran. **B**, **C** show model fits for a recent outbreak at or around the time of data collection for Tizi Ouzou and Jijel. **D**, **E** each shows a model with a constant force of infection for provinces in the south. Black points and error bars represent the mean and 95% binomial confidence interval of the seroprevalence, respectively. They are plotted at the mean age within each 10-year age group. The sample sizes for the calculation of the error bars are as follows, from youngest to oldest age of patients: **A** 81, 37, 77, 69, 31, 31, 30, 7; **B** 33, 18, 30, 34, 23, 13, 10, NA (only 1 observation), 3; **C** 53, 48, 72, 52, 27, 24, 10, 13, 3; **D** 17, 21, 26, 26, 8, 11, 3, 6; **E** 60, 38, 77, 37, 25, 10, 18, 13, 6. The colored lines and ribbons represent the mean and 95% credible interval of the seroprevalence from the models for each province.

In the univariable analysis, we found statistically significant associations between WNV seropositivity and age group, province of residence, region of residence, residence type, living in an area infested by mosquitoes, and outdoor activity (Table 1). There was no significant difference in seropositivity by sex, blood transfusion status, or living near a water reservoir. While seroprevalence in the northern provinces was relatively similar across age, seroprevalence in the southern provinces increased substantially with age (Fig. 2).

In the multivariable analysis (Table 2), significant risk factors for WNV infection included in the final model were age group, region, and residence type (province of residence was nested within region). The adjusted odds ratios (AORs) of WNV infection increased with each age group from 2.2 (95% CI: 1.2–4.2) among those aged 10–29 years, 4.8 (95% CI: 2.7–9.0) among those aged 30–54 years, 5.8 (95% CI: 2.6–12.8) among those aged 55–64, and 6.5 (95% CI: 3.1–13.7) among those aged 65 years or more, all compared to individuals aged 0–9 years-old. Individuals living in the south had 6.4 (95% CI: 4.6–9.1) times the odds of WNV infection compared to those living in the north, adjusting for age group and residence type. Finally, individuals living in suburban/rural areas had 3.0 (95% CI: 2.2–4.3) times the odds of WNV infection compared to those living in urban areas, adjusting for age group and region.

**Table 2:** Multivariable analysis of risk factors for West Nile virus infection in Algeria, 2017–2018. *N* = 1235

Variables	AOR	(95% CI)	*P* value
Age (*N* = 1232, 99.8%)			
0–9	Ref.		
10–29	2.2	(1.2–4.2)	0.01
30–54	4.8	(2.7–9.0)	2e-7
55–64	5.8	(2.6–12.8)	1e-5
65+	6.5	(3.1–13.7)	6e-7
Region (*N* = 1235, 100%)			
South	6.4	(4.6–9.1)	<2e-16
North	Ref		
Residence type (*N* = 1227, 99.4%)			
Suburban/rural	3.0	(2.2–4.3)	2e-10
Urban	Ref		

Adjusted odds ratios and 95% confidence intervals (CIs) were obtained from a logistic regression model with WNV seropositivity as the outcome and predictors for the following covariates: age group, region, and residence type; 1060 non-missing observations were included in all steps of the stepwise down procedure, while 1224 non-missing observations were included in the final, refitted model. We used 2-tailed tests for all models.

*N* participant number, *CI* confidence interval, *AOR* adjusted odds ratio, *Ref* reference category.

### WNND

**Fig. 3:**
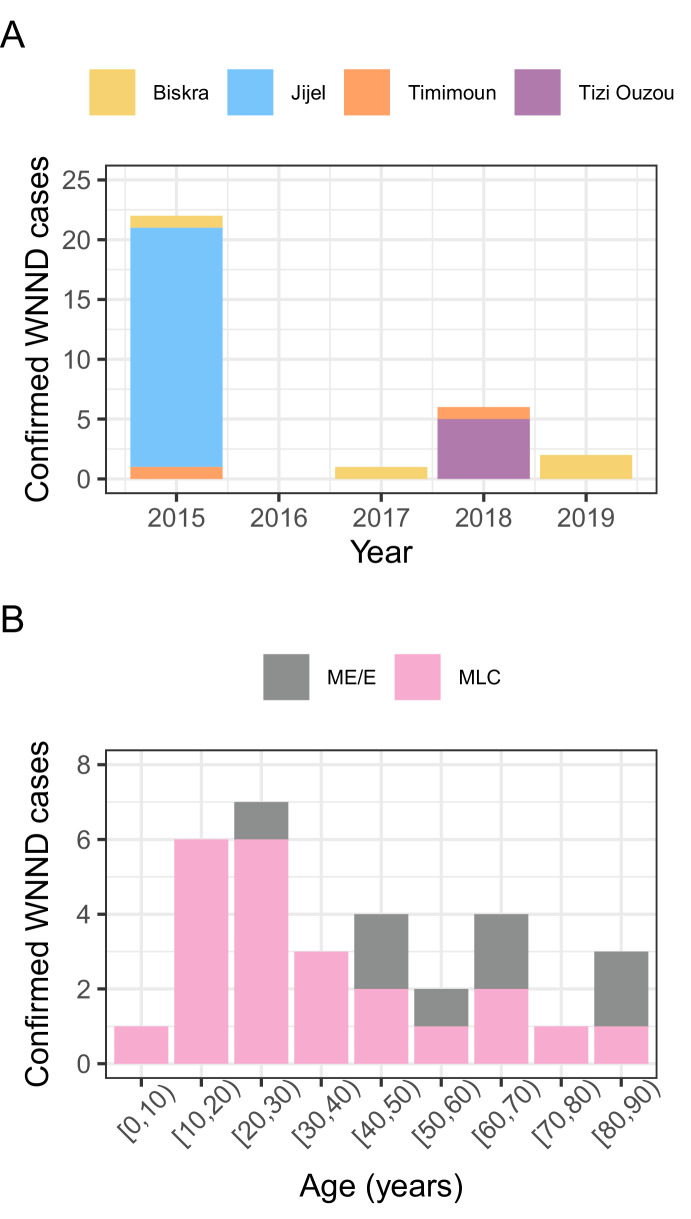
Epidemiology of West Nile neuroinvasive disease (WNND) cases in Algeria, 2015–2019. **A** Confirmed WNND cases by year and province (colored bars). Zero cases were reported in 2016, and zero cases were reported in Oran during the study period. **B** Confirmed WNND cases by age group and clinical form (colored bars). ME/E meningoencephalitis or encephalitis, MLC aseptic meningitis.

Between 2015 and 2019, a total of 479 suspected cases of WNND were reported in the study locations, and of those, 31 cases (6.5%) of WNND were confirmed. While serum samples were available for all patients, some did not have urine or CSF samples, which influenced their diagnostic testing pathway. As summarized in Table S3, most patients tested positive for IgM in serum and/or CSF. These results were often accompanied by a positive IgG test. In four patients with inconclusive serology, follow-up ELISA testing revealed seroconversion. The diagnosis of WNND was further supported by PCR detection of WNV RNA in urine or CSF in 8 cases, and by the identification of neutralizing antibodies through PRNT90 screening in 23 patients. Most confirmed cases of WNND were detected in 2015 or 2018, with 22 and 6 cases, respectively. No WNND cases were recorded in 2016, and only a few cases were detected in 2017 and 2019 (Tables 3 and S3). Patient ages ranged from 6 to 89 years, with a median of 35. The sex ratio was 1.22 males to females. Twenty-three (74.1%) patients had aseptic meningitis, and 8 (25.8%) had ME/E (Tables 3 and S3). Patients with ME/E were significantly older than those with meningitis (*p* = 0.007) (Fig. 3 and Table S3). Although most patients with ME/E were males, there was no significant association between sex and clinical forms (*p* = 0.06, Fig. S2). We report a single death following WNV ME/E from 2015-2019, corresponding to a case fatality rate of 3.2% (1/31) (Tables 3 and S3). Patients were mainly located in the north (*N* = 25 versus *N* = 6 in the south, Fig. 3).

**Table 3:** Cases of neuro-meningeal West Nile virus (WNV) infections in the study locations from 2015 to 2019, according to the clinical forms

WNND	2015	2016	2017	2018	2019	Total	Age years, median (min–max)	Sex ratio (M/F)	Case fatality rate
WNV suspected case (N)	100	63	99	92	125	479	23.9 (5–89)	1.58	1
WNV aseptic meningitis (N)	20	0	0	2	1	23	27 (6–89)	0.77	0
WNV meningo-encephalitis or encephalitis (N)	2	0	1	4	1	8	61 (29–82)	7	1
Total WNND [N (%)]	22 (22%)	0 (0%)	1 (1%)	6 (6.5%)	2 (1.6%)	31 (6.5%)	35 (6–89)	1.22	1(3.22)

*WNND* West Nile Neuroinvasive Disease, *WNV* West Nile Virus, *N* number of cases, *M/F* male to female ratio.

### Serocatalytic models

For the two provinces in the south, Timimoun and Biskra, two models fitted the data well (i.e., had the lowest Pareto-smoothed importance sampling leave-one-out cross-validation (PSIS-LOO)): the constant model and the constant with outbreak model. Given the small differences in expected log pointwise predictive density (ELPD) (<1), we considered the most parsimonious model to be the one with the best fit, which was the constant model, for both locations (Table S4). In this model, the annual probability of infection, which is constant over time, was nearly twice as large in Timimoun (2.2%, 95% credible interval [CrI] 1.6%–2.8%) than in Biskra (1.3%, 95% CrI 1.1%–1.6%) (Table 4). Under these estimates, our model expects that seroprevalence is 23% (95% CrI: 19–28) at 20 years old and 41% (95% CrI: 34–48) at 40 years old in Biskra (Fig. 2). In Timimoun, the model expects 35% (95% CrI: 28–43) seroprevalence at 20 years old and 58% (95% CrI: 48–68) at 40 years old. The one outbreak model had the worst fit to the data (i.e., highest PSIS-LOO).

**Table 4:** Parameters estimated from serocatalytic models fitted to cross-sectional seroprevalence data on West Nile virus in Algeria

Province	Year of sample collection	Model	Attack rate, mean % (95% CrI)	Estimated time of outbreak, mean year (95% CrI)	Annual probability of infection, mean % (95% CrI)
Oran	2018	One outbreak	14 (11–18)	2010 (2008–2012)	-
Tizi Ouzou	2018	Recent outbreak	7.2 (3.8–11)	-	-
Jijel	2017	Recent outbreak	10 (7.1–14)	-	-
Timimoun	2018	Constant FOI	-	-	2.2 (1.6–2.8)
Biskra	2018	Constant FOI	-	-	1.3 (1.1–1.6)

The estimated time of outbreak was rounded to the nearest year.

*CrI* credible interval, *FOI* force of infection.

For Oran in the north, the best-fitting model with the lowest PSIS-LOO was the outbreak model (Table S4). We estimated that an outbreak occurred in 2010 (95% CrI 2008–2012) and infected 14% of the population (95% CrI: 11%–18%) (Table 4). For the other two provinces in the north, Jijel and Tizi Ouzou, the model with the lowest PSIS-LOO and the fewest number of parameters was the recent outbreak model (Table 4 and Supplementary results). Outbreaks may have occurred in these locations around the time of the data collection (2017 for Jijel and 2018 for Tizi Ouzou). We estimated similar attack rates of 7.2% (95% CrI: 3.8%–11%) and 10% (95% CrI: 7.1%–14%) in Tizi Ouzou and Jijel, respectively (Table 4).

To further investigate the timing of outbreaks in the northern provinces, we plotted the seroprevalence by one-year age group for WNV in children (Fig. S3) and indicated the estimated outbreak time. Increases in seroprevalence correspond with the estimated outbreak time for Oran. There is no clear increase in seroprevalence for Jijel or Tizi Ouzou, suggesting children of all ages may have been equally exposed to the virus during a recent outbreak.

All models had good mixing of Markov chain Monte Carlo (MCMC) traces and no warnings about low effective sample sizes. All models except one had R-hat values for the parameters below 1.01. However, some models had divergent transitions after warm-up and Pareto *k* diagnostic values that were too high. Full model diagnostic information can be found in Table S4.

## Discussion

Our study focused on the epidemiology of WNV infection, including transmission patterns and neuroinvasive forms of the disease, in the north and south of Algeria during the late 2010s. This study presents serological evidence that WNV has likely been endemic for decades in the south but emerged only recently in the north of the country, coinciding with climatic^32^ and socio-environmental changes^39,40^.

We found different patterns of historical WNV circulation between the southern and northern provinces of Algeria. Our results showed that seropositivity plateaued at approximately 60% for individuals living in the south aged 40 years and older. A potential explanation for this result is seroreversion; however, we found poor evidence of waning immunity in our study. A more likely alternative explanation is that the virus was introduced into this region approximately 40 years before the samples were collected. This hypothesis is supported by evidence from other studies, including one that demonstrated the first serological evidence of WNV in humans in Djanet (southeast Algeria) in 1973 (44 years before the survey)^34^. Our results are also consistent with the sporadic reports of WNND in the south (2015, 2017–2019). In contrast, we estimated that outbreaks in the north of the country occurred within the last 10–15 years, coinciding with notable climatic shifts^32^. An outbreak may have occurred in Oran in 2010, and in Jijel and Tizi Ouzou near the time of data collection in 2017 and 2018, respectively. The distance between Oran and the eastern provinces (Jijel and Tizi Ouzou) is over 400 km, suggesting independent outbreaks. However, the proximity of Tizi Ouzou and Jijel (150 km apart) may explain why outbreaks in those provinces may have happened around the same period.

Our modeling results are consistent with our epidemiological surveillance, which captured 20 WNND in Jijel in 2015, just two years before data collection for our study began (also, seroprevalence was 0 for children between 1 and 3 years old). They are also consistent with WNND cases reported in Tizi Ouzou in 2018. However, other evidence of transmission of WNV in the north from the literature is less consistent with our findings. There was a fatal human case of WNND in Jijel in 2012^34^, but no further details were available about this case, including recent travel history. Also, a 2012 study found seroprevalence of 6.7% for WNV in Algiers (the capital of Algeria, on the Mediterranean coast, not far from Tizi Ouzou), which is a few years before the recent outbreak we estimated. It is important to note that Algiers has a higher population density and greater travel networks compared to Tizi Ouzou, which could explain the heterogeneity in outbreak or introduction timing^34^. Although WNV transmission is higher in the south than in the north, approximately four times more cases of WNND were reported in the north than in the south during the study period. This finding could be due to differences in the sizes of the surveilled populations. There were 4.3 times more individuals living in the three northern provinces compared to the two southern provinces (3.2 million versus 750,000) during the 2008 Census^41^. Another potential explanation is that detection rates are higher in the north than in the south.

Profound demographic and environmental transformations, driven by demographic growth and socio-economic changes, have occurred in Algeria’s cities and rural areas in recent decades^39,40^. In the north, dense human settlements, irrigation systems, wetlands, and rapid urban development have created favorable conditions for mosquito breeding and development^42^. Algeria has also experienced major climatic changes^32^, including rising temperatures and decreased rainfall, especially in the north, which have likely affected vector ecology and transmission of viruses^4,23,24^. These combined factors have led to the spread of WNV into new areas. Indeed, previous studies conducted in Europe, especially in the Mediterranean region, have shown an association between climate change and the rise in human WNV cases^18^, which could be attributed to the expansion of *Culex* species and an increase in their ability to transmit the virus^23^.

Comparing the seroprevalence of arbovirus infections across different countries is challenging due to variations in sampled populations, laboratory techniques, and study periods (ranging across 21 years). In our study, we found a seroprevalence of 16.7%, which is higher than that of some neighboring countries in the Mediterranean region, such as Libya (2.75% in 2013)^43^, Morocco (3.29% in 2019)^44^, southern France (1.2% in 2019–2020)^45^, Spain (0.13% in 2010–2011)^46^, and Greece (1.5% in 2012–2013)^47^. Higher seroprevalence estimates were reported in Egypt (24% in 1999–2002)^48^ and Tunisia (19.6% in 2017–2020)^49^. Whether study participants lived in rural or urban locations was not always clear in these studies. Three studies (Libya, Morocco, and Spain) did not include children, and it was unclear whether children were included in the studies in France and Tunisia. The Greek study was the only nationally representative study with a large sample size, while the study in Morocco had the smallest sample size with less than 100 individuals across two locations. The seroprevalence estimate from Egypt is similar to that for Biskra in our study (24% vs. 29.1%). One study (in Libya) used ELISA alone to confirm WNV serostatus, which can lead to higher rates of false positives compared to using ELISA followed by PRNT. Differences in seroprevalence of WNV across locations may also be due to differences in human behavior, environmental conditions, and vector control interventions, which affect the rate of human exposure to infected mosquitoes.

We found that the seroprevalence of WNV significantly increased with age in the southern provinces. This finding aligns with previous studies and may be explained by cumulative exposure to the virus over time and the more time spent outdoors by older people compared to children^47^. Residence in the south was also significantly associated with seropositivity, which agrees with previous serological surveys conducted in Algeria^34^. The increased risk of WNV infection in the south could be due to the increased temperature^4^ and the oasis effect. The presence of stagnant water sources, vegetation, and milder temperatures in the oases attracts migratory birds, vectors, and humans, amplifying the transmission cycle of WNV^50^. Differences in seropositivity may also be due to different strains of WNV, which exhibit distinct eco-epidemiological evolutionary characteristics. The strain circulating in the south belongs to the L1 lineage^24^; however, no strain/variant data are available for the north. Our study also demonstrated that the likelihood of being seropositive to WNV is higher among subjects living in rural or suburban areas than in urban areas. One possible explanation for this finding is that cities and villages with dwellings surrounded by vegetation offer an optimal environment for WNV transmission^51^.

One limitation of this study is the small sample size for some age groups (especially those 60 years and over). Larger sample sizes could help reduce uncertainty in our parameter estimates and better characterize the time of viral emergence in the south. Among the best-fitting models for the five provinces, three models had some divergent transitions after warmup, and one model had 8% of Pareto *k* diagnostic values that were too high, even after moment matching. Further data and/or investigation may be warranted given these indications of a lack of fit. Another potential limitation is that the sampling strategy based on recruiting subjects directly from medical analysis laboratories may not have been random and could have introduced selection bias. However, this methodology has been successfully used in several other sero-epidemiological surveys^52^. On the other hand, a strength of our study is that we used PRNT, a gold standard technique. Despite its high cost, time, and equipment requirements, this method was crucial for confirming ELISA results, thereby reducing cross-reactivity with antibodies against other flaviviruses^53,54^ that circulate in the region, such as Usutu virus^55^. Since specific tests for Usutu virus, which belongs to the same serocomplex as WNV and can cross-react even with PRNT, are not available in the country, we did not perform differential tests. However, recent studies indicate that Usutu virus circulation in Algeria is limited and is unlikely to have substantially influenced our results^56,57^.

To gain more insights into viral circulation and transmission patterns, epidemiological surveillance studies in Algeria should continue monitoring WNV cases, particularly in the northern region, and be expanded to more provinces. In addition, research should be conducted to investigate potential factors that contribute to WNV spread in the country, including the impact of climate change, the transmission ability of vectors, and the genetic characterization of circulating lineages.

## Methods

### Ethical approval

The seroprevalence study was conducted in accordance with national and institutional guidelines and regulations, and was approved by the Ethics committee of Blida 1 University’s faculty of medicine in Blida, Algeria (01/CEEC/2025). The Ethics committee determined that verbal consent from patients was appropriate, as the activity fell under routine public health surveillance and used leftover sera.

Samples from patients with suspected viral neuroinvasive diseases were included in a national diagnostic protocol as part of the Algerian Ministry of Health surveillance program for WNV infections and were submitted for diagnosis to the National Reference Center for Arboviruses and Emerging Viruses. Hence, informed consent was not necessary.

Participants did not receive compensation for this study.

### Study design and sample collection

A cross-sectional seroprevalence study was carried out in five provinces (wilayas) in Algeria. The provinces were selected using a tool developed by the European Center for Disease Prevention and Control that assesses the risk of WNV circulation based on factors, such as wetlands, the presence of migratory bird stopover sites, and previous WNV detection in animals, humans, or vectors (Table S1)^58^. The study population consisted of individuals living in urban, suburban, or rural areas within the selected provinces. The study locations included two high-risk areas (Jijel in the northeast and Timimoun in the southwest, with 636,731 and 33,059 inhabitants, respectively), one intermediate-risk area (Biskra in the southeast, with 721,051 inhabitants), and two low-risk areas (Oran in the northwest and Tizi Ouzou in the north central, with 1,452,317 and 1,125,664 inhabitants, respectively) (Fig. 1)^41^.

For the seroprevalence study, the required sample size was estimated at a minimum of 800 individuals, with an average prevalence of 15% based on seroprevalence data in North African and Mediterranean countries^28,29,34,47^,a  precision of 2.5%, and a 95% confidence interval (CI).

Leftover serum samples were collected from public and private laboratories from patients either seeking routine checkup analyses (e.g., blood tests for cholesterol, blood glucose, thyroid function, vitamin levels) or displaying different pathologies with no bias toward a particular set of symptoms. All samples were collected throughout the year in 2018, except those from Jijel, which were collected in 2017. Eligibility criteria included residing in the study area for at least one year prior to enrollment. Sera from subjects with immunological disorders or ongoing infectious disease (which required immunological, microbiological, or virological investigations) or those presenting with fever were discarded to reduce bias. Participating laboratories recorded basic socio-demographic information (i.e., age, sex, location, and type of residence) and risk factors of exposure to WNV infection^59^ (i.e., blood transfusion, self-reported residence in a mosquito-infested area, residence near a water reservoir, main activities carried out outdoors) at the time of specimen collection. Sex was determined by a healthcare practitioner. Information about gender was not available for our study.

For the detection of human cases of WNND, the investigation focused on hospitalized patients residing in any of the five study locations presented with aseptic meningitis or ME/E between June 1st and October 31st each year from 2015 to 2019. In line with the Algerian Ministry of Health surveillance framework, a suspected case of WNND was defined as a patient exhibiting neurological manifestations (e.g., aseptic meningitis or meningoencephalitis), along with fever (≥38.5 ^ᵒ^C) and cerebrospinal fluid (CSF) showing a lymphocytic predominance (>50%) and normal glycorrhachia, where a viral etiology could not be excluded.

According to the national surveillance plan, recommended biological samples for laboratory confirmation of WNND cases were submitted to the National Reference Center for Arbovirus and Emerging Viruses (Institut Pasteur d’Algérie). A case report form was requested for each suspected case. Samples, including CSF and serum taken at the time of hospitalization for neurological symptoms (acute samples) and urine taken within four weeks after symptom onset, were tested for WNV RNA, IgM, and/or IgG antibodies. Additional follow-up serum samples were collected after day 8 of illness from some patients whose initial serology was inconclusive to demonstrate specific IgG seroconversion. A confirmed case of WNND met at least one of the following laboratory criteria: (i) detection of WNV-specific IgM antibody in CSF by ELISA, (ii) detection of WNV RNA in urine and/or CSF by PCR, or (iii) detection of IgM and IgG antibodies in serum by ELISA confirmed by detection of WNV neutralizing antibodies using the plaque reduction neutralization test (PRNT). Further details about the diagnostic testing algorithm can be found in Supplementary methods and Fig. S1.

### Laboratory methods

*WNV seroprevalence study*. Sera were tested for the presence of WNV IgG antibodies by a commercial indirect ELISA test (Anti-WNV IgG ELISA, Euroimmun, Lubeck, Germany) in a Biosafety Level 2 laboratory. The assay was performed according to the manufacturer’s instructions. Samples with an index value of 1.1 and above were considered positive for WNV IgG antibodies. An index value of less than 0.8 meant that IgG antibodies were not detected, while an index value of ≤1.1 and ≥0.8 was considered borderline.

All samples with positive or borderline IgG ELISA results were further analyzed by PRNT to detect specific neutralizing antibodies for WNV. A qualitative PRNT was performed as follows: each heat-inactivated serum (at 56 °C for 30 min) was diluted to 1:20. A volume of 30 µl of each diluted serum was mixed with an equal volume of WNV strain Eg101 (10^3^ Plaque Forming Units (PFU) per mL) and incubated overnight in duplicate in a 96-well plate at 4 °C. In parallel, a virus back-titration was performed to confirm the virus dose and ensure accurate interpretation of neutralization results. A stepwise dilution series was prepared from the virus stock (10^3^ PFU/mL) by sequential dilution in cell maintenance medium (Leibovitz’s L-15 medium (Gibco, USA) supplemented with 2% Newborn Calf Serum). The dilutions were prepared as follows: D50 was obtained by mixing 150 µL of virus stock with 150 µL of medium; D70 by mixing 180 µL of D50 with 120 µL of medium; D90 by mixing 100 µL of D70 with 200 µL of medium; D95 by mixing 150 µL of D90 with 150 µL of medium; and D99 by mixing 60 µL of D95 with 240 µL of medium. For each dilution, 30 µL was added in duplicate to wells (without serum) and mixed with an equal volume of cell maintenance medium. The following day, 30 µL of a suspension of Porcine Stable kidney (PS) cell line (obtained from Institut Pasteur of Dakar, Senegal), previously cultured to confluence at 37 °C in Leibovitz’s L-15 medium supplemented with 10% Newborn Calf Serum (growth medium), was added at 6.10^4^ cells/30 µl/well, followed by incubation for 3 h at 37 °C in 5% CO_2_. An overlay of 60 µl of Carboxymethylcellulose sodium salt (1.6%) was added to each well, and the plates were further incubated at 37 °C in 5% CO_2_ for 4 days. After incubation, cells were fixed with 10% paraformaldehyde for 1 h and stained with amido black solution at room temperature. Each assay included a virus-only control (10^3^ PFU/mL), a cell-only control, and positive and negative serum controls. The number of plaques per well was assessed by virus back-titration. Serums with at least a 90% reduction in plaques (PRNT90) were considered positive compared to virus control. Samples were considered seropositive for WNV if they were positive or borderline for ELISA and positive for PRNT.

*WNND case confirmation*. Laboratory investigation of suspected WNND was carried out by WNV antibody detection and PCR testing. Thus, collected sera and CSF were first analyzed for IgM and IgG antibodies by commercial ELISA kits (anti-WNV IgM and IgG ELISA, Euroimmun). Patients with positive results from ELISA were confirmed by PRNT, as mentioned earlier, and/or by real-time RT-PCR (RT-qPCR) on ABI 7500^Ⓡ^ real-time PCR system (Applied Biosystems, Foster City, USA) for specific detection of WNV RNA in urine and/or CSF samples. The viral RNA was detected using RT-qPCR assays targeting the conserved 5’ non-coding (NC) region and the envelope (*E*) gene^60,61^. The viral nucleic acid was extracted from 140 µL of each sample using QIAmpl Viral RNA Kit (Qiagen, Germany) and eluted in 60 µL of RNase-free water. The RT-qPCR amplification of the 5’ NC region was performed using the SuperScript III Platinum One-Step qRT-PCR Kit (Invitrogen, ThermoFisher Scientific, USA). The reaction was carried out in a total volume of 25 µL, containing 5 µL of RNA, 0.5 µL of SuperScript® III/Platinum® Taq mix, 12.5 μl 2× reaction mix, 0.4 μM of each primer, and 0.2 μM of probe. Cycling conditions were as follows: 50 °C for 30 min, 95 °C for 2 min, 40 cycles of 95 °C for 15 s, and 60 °C for 30 s. Positive samples were additionally analyzed using the *E* gene RT-PCR assay, under the same conditions as described above, with primers and probe adjusted to 0.8 μM and 0.4 μM, respectively. The list of primer and probe sequences used in both assays is provided in Table S2.

### Descriptive analysis

We presented data on demographics and risk factors for WNV infection as frequencies with 95% exact binomial CIs. We performed logistic regression for the univariable analysis to test associations between categorical variables and WNV seropositivity. For each variable, samples with missing information were excluded from the analysis for that variable. We also performed multivariable logistic regression using a stepwise down procedure. All variables with a *p*-value < 0.20 in the univariable analysis (except for outdoor activity, which had over 60% of observations missing) were initially included in the multivariable logistic regression model. Variables with the highest *p*-value were removed from the model one at a time until all remaining variables were statistically significant at the 0.05 level. The multivariable model was fitted to the same observations across all steps of the stepwise down procedure. The final multivariable model was refitted using non-missing observations for only selected covariates. For each variable retained in the final model, AORs were calculated along with their 95% CI. We used 2-tailed tests for all logistic regression models.

The geographical distribution of WNV seroprevalence was visualized using the free and Open Source QGIS software version 3.40.9. All other descriptive analyses were performed using R version 4.3.2 (The R Foundation for Statistical Computing, https://www.r-project.org/).

### Serocatalytic models

The analysis of age-stratified seroprevalence data with serocatalytic models can provide insights into the history of viral circulation^62^. In serocatalytic models, the per-capita rate of new infections among susceptible individuals, known as the force of infection (FOI), can be inferred from age-stratified seroprevalence data for pathogens that elicit a durable or well-characterized immune response. If we let *λ*(*k*) be the instantaneous FOI at age *k*, then the probability that an individual of age *a* has ever been infected is:

1 $$P\left(a\right)=1-\exp (-{\int }_{k=0}^{a}\lambda \left(k\right){{{\rm{dk}}}})$$

Here $-{\int }_{k=0}^{a}\lambda \left(k\right)\,{{{\rm{dk}}}}$ is the cumulative hazard of infection from birth to age *a*. In the special case of a constant FOI, this simplifies to:

2 $$P\left(a\right)=1-\exp \left(-\lambda a\right)$$

We can derive the annual probability of infection from serocatalytic models as 1-exp(-FOI). For each study location, we used the Rsero package^63^ in R to fit five models representing different patterns of transmission to the seroprevalence data (not the WNND data). The models were: 1. constant FOI (“constant”), 2. constant FOI and seroreversion (“constant with seroreversion”), 3. constant FOI with occasional outbreaks (“constant with outbreak”), 4. one outbreak, and 5. a recent outbreak.

The constant model is one of the simplest models we considered, with a single parameter, the FOI, which is assumed to be constant. In the seroreversion model, individuals who have seroconverted face an annual probability of seroreverting to seronegative, which captures waning immunity. For the outbreak and constant with outbreak models, the FOI is estimated as a sum of *K* Gaussian distributions, where *K* is the number of outbreaks. The constant with outbreak model has a constant FOI (like the constant model), but one or more outbreaks can still occur^64^. Like the constant model, the recent outbreak model has a single parameter, the attack rate. It can be interpreted as an outbreak that occurred roughly around the time of the survey and infected all age groups equally. Model equations can be found in the Supplementary Methods.

Rsero uses the rstan R package (version 2.32.3)^65^ to estimate model parameters. Model convergence was assessed by visually inspecting MCMC traces, ensuring that the potential scale reduction factor, R-hat, was below 1.01 for all parameters, checking that the number of divergent transitions was low, and verifying that the effective sample sizes were at least 400 (for four chains)^66^. Further details about model fitting can be found in Supplementary Methods.

Model selection was based on PSIS-LOO, with the best-fitting model having the lowest PSIS-LOO^67^. PSIS-LOO is the recommended model comparison criterion for Stan^68^. For models with similar PSIS-LOO values, the most parsimonious model is preferred. We also computed the difference in ELPD and the standard error of the ELPD to provide an additional level of support for models being similar or different. If the difference in ELPD is less than 4, the difference between models is considered small^69^. We checked that the Pareto *k* diagnostic values, which monitor the sampling accuracy of LOO, were low. If more than 1% of Pareto *k* values were classified as too high, we performed a moment matching correction to the importance sampling for the problematic observations to improve the accuracy of the result^70^. We did this by setting the argument moment_match to TRUE in the loo() function. Very high Pareto *k* values typically suggest the model is mis-specified or that there are outliers or mistakes in data processing^68^. Model fitting and visualization were performed using R version 4.3.2 (The R Foundation for Statistical Computing, https://www.r-project.org/).

### Reporting summary

Further information on research design is available in the Nature Portfolio Reporting Summary linked to this article.

## Supplementary information

Supplementary Information

Peer Review File

Reporting Summary

## Source data

Source Data

## Data Availability

The aggregated seroprevalence data, including sample year, province, PRNT90 result (positive or negative), and age in 10-year groups, generated in this study have been deposited in the Zenodo database under accession code 10.5281/zenodo.20116899 (10.5281/zenodo.20116898) and are available on GitHub (https://github.com/kcharniga/wnv_algeria). The raw seroprevalence data are protected and are not available due to data privacy laws. Researchers interested in accessing the study data are encouraged to submit a formal request to either the corresponding author (AH, ahachid@pasteur.dz or hachidaissam1@gmail.com) or his laboratory (email: laboratoire.arbovirus@pasteur.dz, phone: +213 20 32 23 62). To uphold ethical standards and ensure appropriate data use, each request will undergo a case-by-case review and approval process. Additionally, as the data include information collected in Algeria, access is subject to Algerian data ownership regulations and may require approval from relevant Algerian authorities. Final approval is expected within two months, and if access is granted, the duration of data access will be granted on a case-by-case basis. The data generated in this study for Fig. 3 are provided in the Source Data file. Data on WNND cases can be found in Table S3. Source data are provided with this paper.

## Code availability

Code to perform the descriptive analysis and run the serocatalytic models is available on GitHub (https://github.com/kcharniga/wnv_algeria).
