# Supplementary material for: Patterns of emergence and circulation of West Nile Virus in Algeria
Source: Nat Commun. 2026 Jul 6;17:8370. doi: 10.1038/s41467-026-74856-6 (PMC13473090; doi:10.1038/s41467-026-74856-6)
Supplement: Supplementary file 1 [file 41467_2026_74856_MOESM1_ESM.pdf]

Supplementary Information: Patterns of Emergence and Circulation of West Nile Virus in Algeria

Contents

Supplementary methods 2
Supplementary results 4
Supplementary figures 5
Supplementary tables 9

Supplementary methods

Model equations. Serocatalytic models were run using the current version of the Rsero package (https://doi.org/10.1371/journal.pcbi.1012777). The following corresponds to the model equations used in this analysis.

Constant model: the probability that an individual of age *a* is seropositive is $P = 1 - \exp(-a\lambda)$, where λ is the force of infection (FOI).

Constant model with seroreversion: $P = \frac{\lambda}{\lambda+\rho}(1 - e^{-a(\lambda+\rho)})$, where ρ is the seroreversion rate.

Outbreak model: the outbreak model is described as the sum of *K* Gaussian distributions, where K is the number of epidemics. The FOI in year *i* is $\lambda_i = \sum_{k=1}^{K} \bar{\alpha}_k \exp\left(-(i - T_k)^2\right)$, where $\bar{\alpha}_k = \alpha_k \frac{1}{\sum_{i=0}^{A} \exp\left(-(i-T_k)^2\right)}$. Here, T_k is the peak year of the focal epidemic and 1-exp(-α_k) is the attack rate of the epidemic.

Constant with outbreak model: in this model, the FOI is constant, but one or more outbreaks can still occur. The FOI during year *i* is $\lambda_i = \lambda_C + \sum_{k=1}^{K} \bar{\alpha}_k \exp\left(-(i - T_k)^2\right)$, where λ_C is the constant component of the FOI.

Recent outbreak model: in this model, an outbreak occurred at or around the time of the serosurvey and infected everyone equally. Thus, the FOI is independent of age and the probability that an individual is seropositive is $P = 1 - \exp(-\lambda)$.

Bayesian methods. For each model (except for the constant with outbreak model for Oran and Jijel), we ran four chains of 5,000 iterations with a burn-in of 2,500. We used the defaults for the remaining settings in stan (i.e., thin = 1, adapt_delta = 0.95, max_treedepth = 10). For the constant with outbreak model in Oran and Jijel, we ran four chains of 10,000 iterations with a burn-in of 5,000.

Prior distributions. We used Rsero's default prior distributions for all parameters. Rsero uses a non-centered parameterization for the parameters when a normal or lognormal prior distribution

is specified. Non-centered parameterization is used to improve model efficiency, namely to increase the effective sample size for the same number of iterations and to improve convergence (https://mc-stan.org/docs/2_18/stan-users-guide/reparameterization-section.html). The default prior distributions for each of the parameters are as follows: for the outbreak and constant with outbreak models, the attack rates (α) are estimated with lognormal prior distributions as

$$\alpha_j = \alpha_j^1 * \exp\left(\alpha_j^2 * \alpha_{j,raw}\right) \quad (1)$$

Where $\alpha_{j,raw} \sim N(0,1)$ and $\alpha_j^1 = 0.2$ and $\alpha_j^2 = 0.2$.

Non-centered parameterization is used for the peak times of the outbreak, T_j, as

$$T_j = T_j^1 + T_j^2 * T_{j,raw} \quad (2)$$

Where $T_{j,raw} \sim N(0,1)$ and $T_j^1 = 20$ and $T_j^2 = 10$. Note that the default values for T_j do not preclude negative values for outbreak time; negative values would suggest outbreaks in the future which does not make sense given the cross-sectional study design. The prior distribution of the FOI for the constant model is $\lambda = \lambda^1 * \exp(\lambda^2 * \lambda_{raw})$ where $\lambda^1 = 0.01$ and $\lambda^2 = 1$ by default. The default prior distribution for the seroreversion rate is $\rho = \rho^1 * \exp\left(\rho^2 * \rho_{raw}\right)$ where $\rho^1 = 1$ and $\rho^2 = 1$.

For the recent outbreak model, the prior distribution of the attack rate is beta with α (shape 1) = 1 and β (shape 2) = 1.

As mentioned in the main text, we tried two alternative prior distributions for the outbreak timing parameter for the outbreak models in Tizi Ouzou and Jijel. They are shown in Figure S3 (panels B and C).

Diagnostic algorithm for WNND. When a patient is suspected of having WNND, the first step is to test serum for West Nile virus-specific IgM using ELISA (Figure S1). If this test is positive, further confirmation tests, including those for IgG in serum, IgM in cerebrospinal fluid (CSF), and/or PCR (in urine and/or CSF), as well as PRNT90 in serum, may be required depending on

which samples are available. If IgM is not initially detected in serum, a follow-up ELISA is required on serum collected after at least day 8 of illness onset to assess seroconversion. A WNND case is confirmed if at least one of the following is true: (i) WNV-specific IgM antibodies are detected in CSF, (ii) WNV RNA is detected in urine and/or CSF by PCR, or (iii) IgM and IgG antibodies are detected in serum by ELISA followed by detection of WNV neutralizing antibodies by PRNT90. If none of the criteria are met, an alternative diagnosis should be explored. Note that when CSF volume is limited, PCR is prioritized over IgM ELISA; otherwise, PCR is performed regardless of IgM CSF results for research purposes (e.g., strain genotyping).

Supplementary results

One outbreak models for Jijel and Tizi Ouzou. For both Jijel and Tizi Ouzou, the one outbreak model also had low PSIS-LOO values (with differences in ELPD of <4 compared to the recent outbreak model). However, the 95% CrI for the outbreak timing parameter in the outbreak model for both provinces included negative values using the default prior distributions, which means the estimated outbreak time was in the future. For both provinces, using an exponential distribution fixed the problem of estimating outbreak times in the future but greatly increased the number of divergent transitions by an order of magnitude. Initialization of the Markov chain Monte Carlo algorithm failed for both provinces using an alternative normal distribution (Figure S4).

Supplementary figures

Figure S1. Flow chart of the diagnostic algorithm for suspected cases of West Nile Neuroinvasive Disease (WNND).

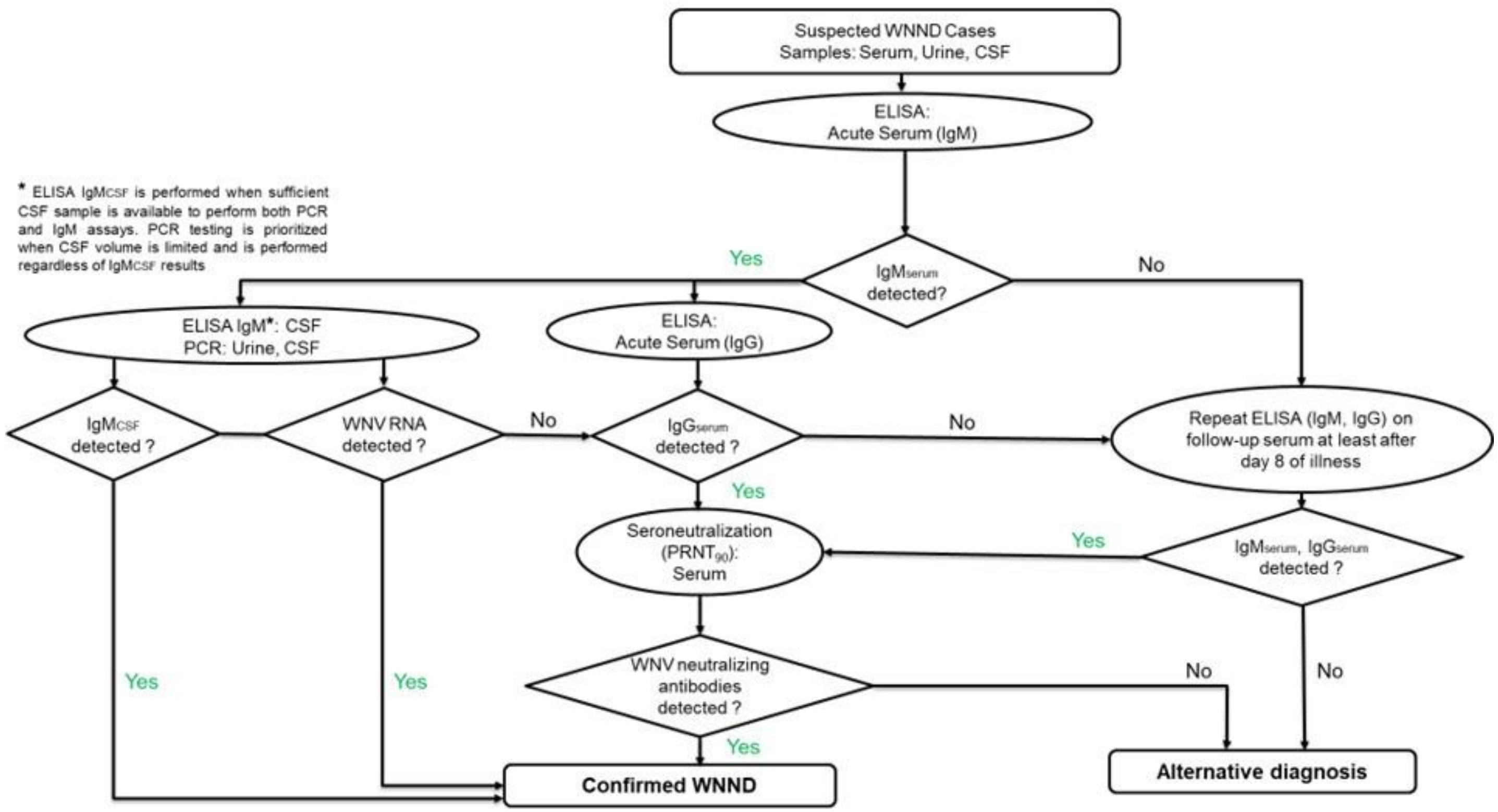

Figure S2. Confirmed West Nile Neuroinvasive Disease (WNND) cases in Algeria, 2015 – 2019 by sex and disease form. N= 31. Color indicates clinical form. ME/E: meningo-encephalitis or encephalitis, MLC: aseptic meningitis.

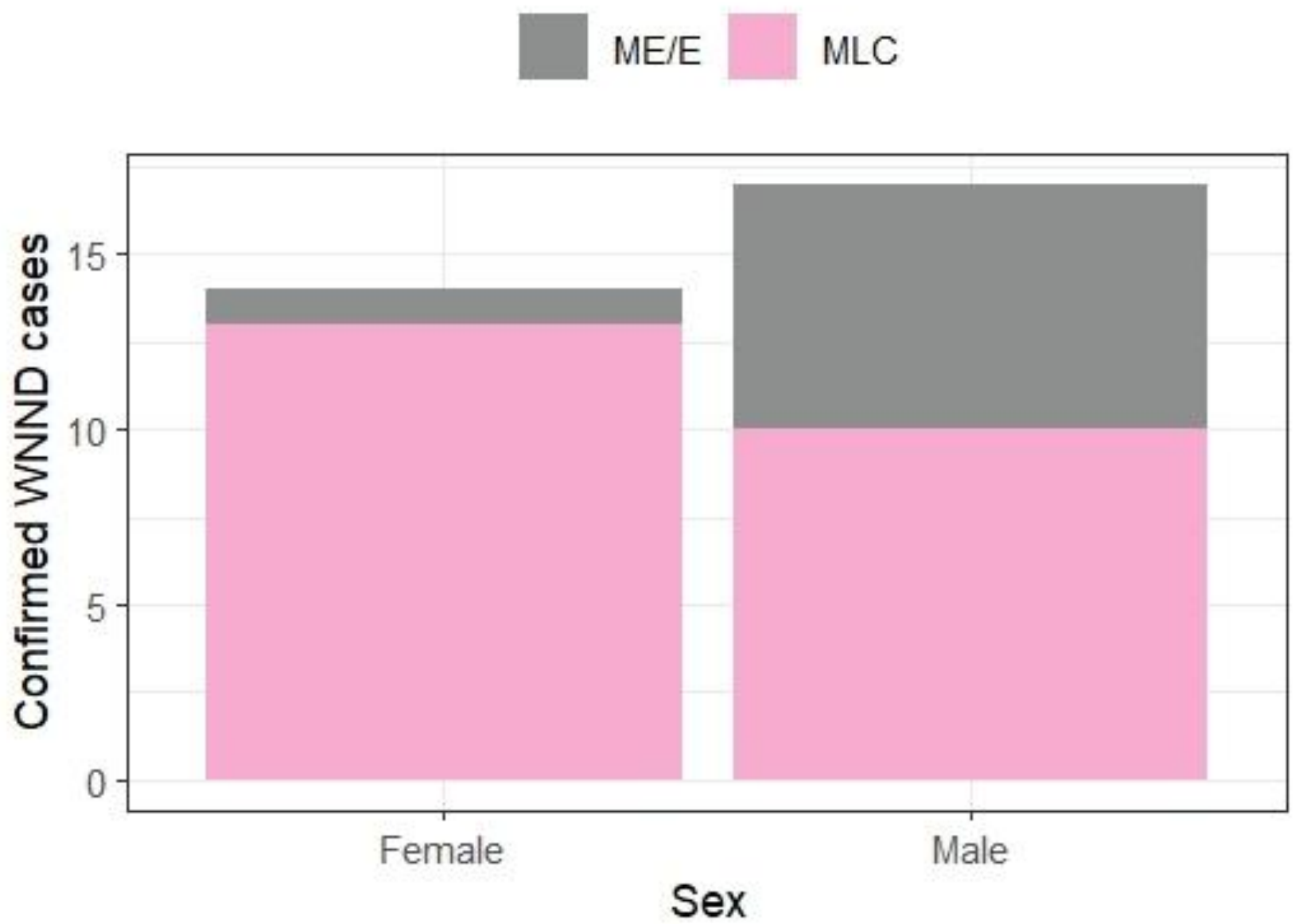

Figure S3. Seropositivity (points) and 95% binomial confidence intervals (error bars) of West Nile virus among children under 13 years of age across three northern Algerian provinces in 2017-2018. Missing points and error bars for Tizi Ouzou mean no children of that age were sampled in that province. Colors come from the best-fitting models: purple color represents the ages of children who were not exposed to an outbreak (“None”), while pink color corresponds to the ages of children during the estimated outbreak time, which was rounded to the nearest year and includes the 95% credible intervals for Oran. For recent outbreaks in Jijel and Tizi Ouzou, every point and error bar is colored pink. Sample sizes for this figure are 97, 39, and 65 for Oran, Tizi Ouzou, and Jijel, respectively.

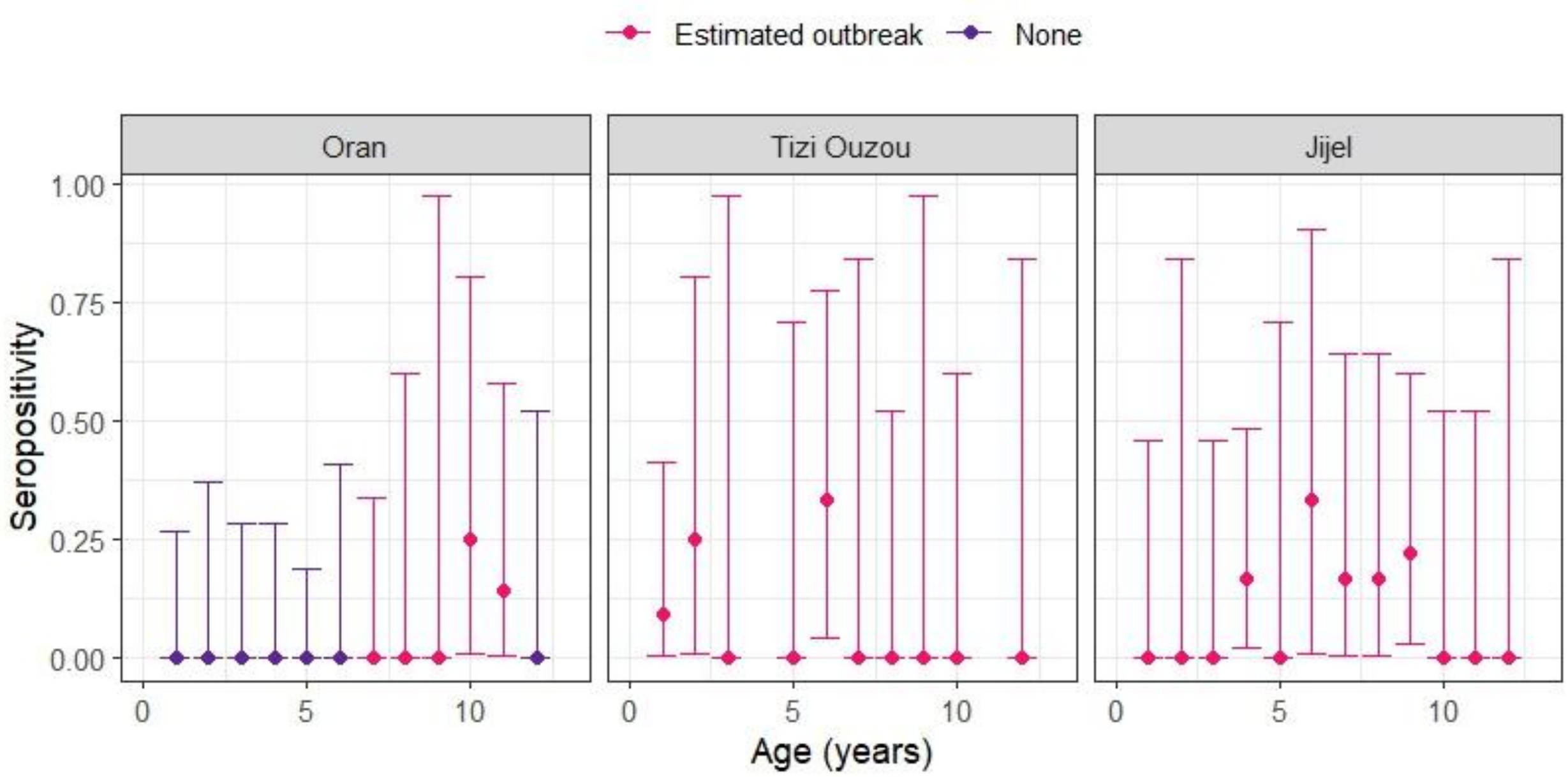

Figure S4. Alternative prior distributions for the outbreak timing parameter in the outbreak models for Jijel and Tizi Ouzou. A. shows Rsero's default prior distribution which includes negative values. B. and C. show the two alternative prior distributions that we tried. For A and B, a non-centered parameterization is used; the normal distribution has mean 0 and standard deviation 1.

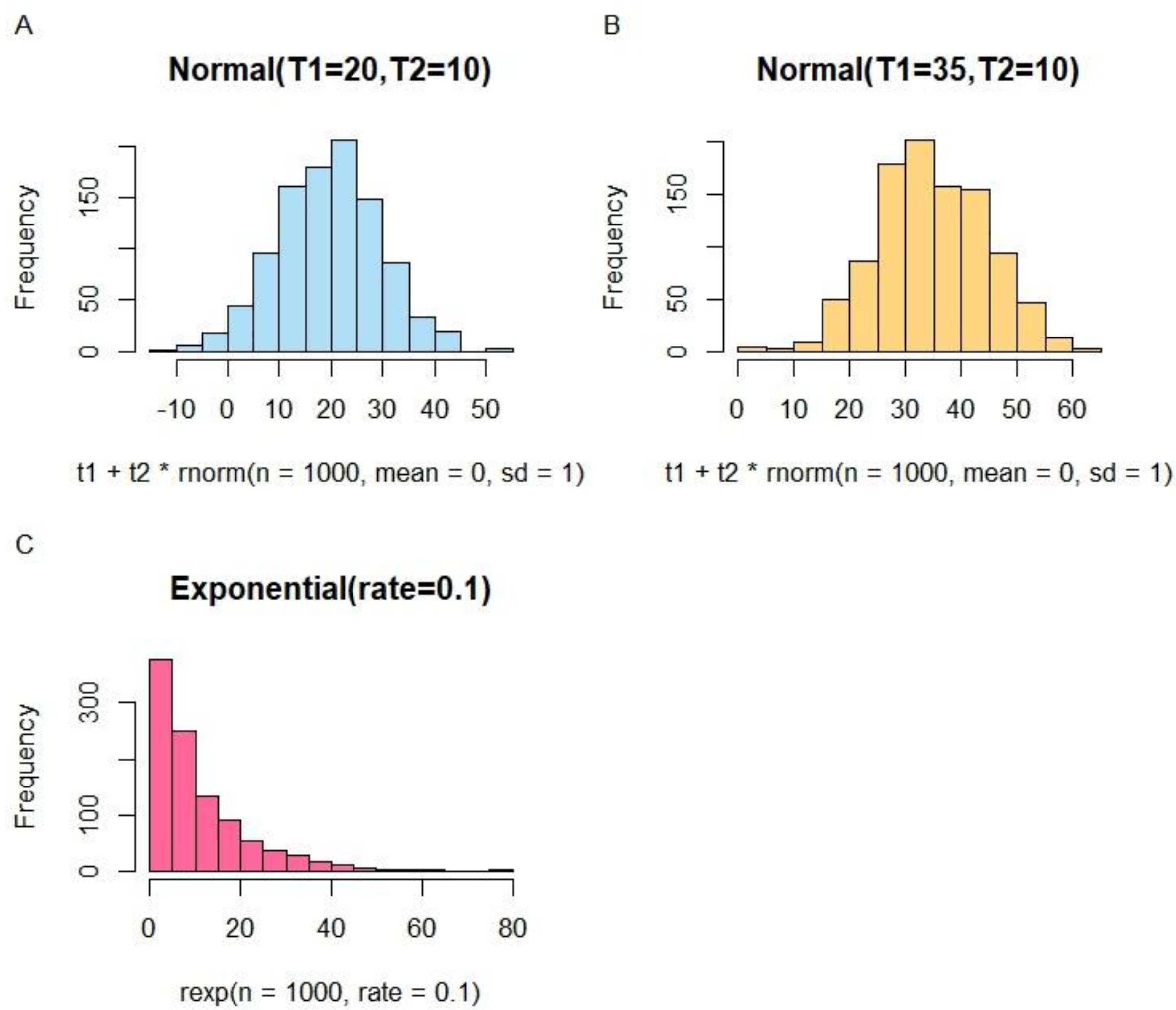

Supplementary tables

Table S1. Study locations based on the risk assessment of West Nile virus circulation using a tool developed by the European Centre for Disease Prevention and Control.

Province	Risk area type	Confirmed case	Seroprevalence study	Ecological factors	Risk assessment	Description
Oran	Predisposed	-	-	+	Low	Ecological conditions suitable for WNV circulation but no historical circulation of WNV
Tizi Ouzou	Predisposed	-	-	+	Low	Ecological conditions suitable for WNV circulation but no historical circulation of WNV
Biskra	Imperiled	-	+	+	Intermediate	Past evidence of WNV circulation
Jijel	Affected	+	-	+	High	Detection of at least one human case according to the European Union case definition.
Timimoun	Affected	+	+	+	High	Detection of at least one human case according to the European Union case definition.

Table S2. Primers and probes for a West Nile virus-specific real-time polymerase chain reaction (PCR) used in this study.

Oligo name	Primer and probe sequences (5'→3')	Target	Position	Reference
ProC-F1	CCTGTGTGAGCTGACAAACTTAGT	5' NC region	10–33	Linke, S., Ellerbrok, H., Niedrig, M., Nitsche, A. & Pauli, G. Detection of West Nile virus lineages 1 and 2 by real-time PCR. *Journal of Virological Methods* **146**, 355–358 (2007).
ProC-R	GCGTTTTAGCATATTGACAGCC		132–153	
ProC-TM*	6FAM-CCTGGTTTCTTAGACATCGAGATCTXCGTGCP		89–113	
WN-LCV-F1	GTGATCCATGTAAGCCCTCAGAA	*E* gene	10597–10619	Del Amo, J. *et al.* A novel quantitative multiplex real-time RT-PCR for the simultaneous detection and differentiation of West Nile virus lineages 1 and 2, and of Usutu virus. *Journal of Virological Methods* **189**, 321–327 (2013).
WN-LCV-R1	GTCTGACATTGGGCTTTGAAGTTA		10649–10672	
WN-LCV-S1	FAM-AGGACCCCACATGTT-MGB		10633–10647	
WN-LCV-S2	VIC-AGGACCCCACGTGCT-MGB		10633–10647	

* X: 5-carboxytetramethylrhodamine (5'-TAMRA) attached to 5'-ethylamino-dThymidin, p: phosphate, FAM: 6-carboxyfluorescein.

Table S3. Virological findings of West Nile virus neuroinvasive disease (WNND) cases (N=31) and case confirmation criteria.

Case	Age group (years)	Sex	Region	Year of illness	Clinical presentation	Testing results of initial samples	Follow-up serum results*	WNND confirmation criteria
1	[40,50)	M	Biskra	2019	AM	IgM_{CSF} +, IgM_S+, IgG_S+, PCR_{CSF} -, PCR_U -, $PRNT_{90}$ +	/	IgM_{CSF} and PRNT
2	[40,50)	M	Biskra	2019	E/ME	IgM_{CSF} +, IgM_S+, IgG_S-, PCR_{CSF} -, PCR_U +	/	IgM_{CSF} and RNA
3	[10,20)	F	Tizi Ouzou	2018	AM	IgM_S+, IgG_S+, $PRNT_{90}$ +	/	IgMs IgGs and PRNT
4	[60,70)	M	Timimoun	2018	E/ME	IgM_{CSF} +, IgM_S+, IgG_S+, PCR_U -, $PRNT_{90}$ +	/	IgM_{CSF} and PRNT
5	[20,30)	F	Tizi Ouzou	2018	AM	IgM_{CSF} +, IgM_S+, IgG_S-, PCR_{CSF} +, PCR_U +	/	IgM_{CSF} and RNA
6	[60,70)	M	Tizi Ouzou	2018	E/ME	IgMs+, IgGs+, PCR_U -, PRNT90 +	/	IgMs IgGs and PRNT
7	[80,90)	M	Tizi Ouzou	2018	E/ME	IgM_{CSF} +, IgM_S+, IgG_S+, PCR_U -, $PRNT_{90}$ +	/	IgM_{CSF} and PRNT
8	[20,30)	M	Tizi Ouzou	2018	E/ME	IgM_{CSF} +, IgM_S+, IgG_S+, PCR_{CSF} +, PCR_U +	/	IgM_{CSF} and RNA
9	[80,90)	M	Biskra	2017	E/ME	IgM_{CSF} +, IgM_S+, IgG_S+, $PRNT_{90}$ +	/	IgM_{CSF} and PRNT
10	[0,10)	F	Biskra	2015	AM	IgM_S+, IgG_S+, $PRNT_{90}$ +	/	IgMs IgGs and PRNT
11	[70,80)	M	Jijel	2015	AM	IgM_S+, IgG_S+, PCR_{CSF} -, $PRNT_{90}$ +	/	IgMs IgGs and PRNT
12	[30,40)	F	Jijel	2015	AM	IgM_S+, IgG_S -, PCR_U +	/	RNA
13	[10,20)	M	Jijel	2015	AM	IgM_S+, IgG_S -, PCR_U -, PCR_{CSF} -	IgM_S+, IgG_S+, $PRNT_{90}$ +	Seroconversion and PRNT
14	[60,70)	F	Jijel	2015	AM	IgM_S+, IgG_S -, PCR_U -, PCR_{CSF} -	IgM_S+, IgG_S+, $PRNT_{90}$ +	Seroconversion and PRNT
15	[10,20)	M	Jijel	2015	AM	IgM_S+, IgG_S -, PCR_{CSF} +, PCR_U -	/	RNA
16	[20,30)	F	Jijel	2015	AM	IgM_S+, IgG_S -, PCR_{CSF} -, PCR_U -	IgM_S+, IgG_S+, $PRNT_{90}$ +	Seroconversion and PRNT
17	[50,60)	F	Jijel	2015	AM	IgM_S+, IgG_S+, PCR_{CSF} -, PCR_U -, $PRNT_{90}$ +	/	IgMs IgGs and PRNT
18	[60,70)	M	Jijel	2015	AM	IgM_S+, IgG_S -, PCR_{CSF} -, PCR_U +	/	RNA
19	[20,30)	F	Jijel	2015	AM	IgM_S+, IgG_S -, PCR_{CSF} -, PCR_U +	/	RNA
20	[10,20)	M	Jijel	2015	AM	IgM_S+, IgG_S+, $PRNT_{90}$ +	/	IgMs IgGs and PRNT
21	[30,40)	M	Jijel	2015	AM	IgM_S+, IgG_S+, PCR_{CSF} -, $PRNT_{90}$ +	/	IgMs IgGs and PRNT
22	[80,90)	F	Jijel	2015	AM	IgM_S+, IgG_S +, PCR_{CSF} +	/	RNA
23	[20,30)	M	Jijel	2015	AM	IgM_S+, IgG_S+, PCR_{CSF} -, $PRNT_{90}$ +	/	IgMs IgGs and PRNT

24	[20,30)	F	Jijel	2015	AM	IgM_S+, IgG_S+, PCR_{CSF} -, $PRNT_{90}$ +	/	IgMs IgGs and PRNT
25	[10,20)	M	Jijel	2015	AM	IgM_S+, IgG_S+, $PRNT_{90}$ +	/	IgMs IgGs and PRNT
26	[40,50)	F	Jijel	2015	AM	IgM_S+, IgG_S+, $PRNT_{90}$ +	/	IgMs IgGs and PRNT
27	[20,30)	F	Jijel	2015	AM	IgM_S+, IgG_S+, $PRNT_{90}$ +	/	IgMs IgGs and PRNT
28	[10,20)	F	Jijel	2015	AM	IgM_S+, IgG_S+, PCR_{CSF} -, PCR_U -, $PRNT_{90}$ +	/	IgMs IgGs and PRNT
29	[20,30)	M	Jijel	2015	AM	IgM_S+, IgG_S+, $PRNT_{90}$ +	/	IgMs IgGs and PRNT
30	[50,60)	F	Jijel	2015	E/ME	IgM_S+, IgG_S -	IgM_S+, IgG_S+, $PRNT_{90}$ +	Seroconvertion and PRNT
31	[40,50)	M	Timimoun	2015	E/ME	IgM_{CSF} +, IgM_S+, IgG_S+, $PRNT_{90}$ +		IgM_{CSF} and PRNT

*: follow-up ELISA was performed on samples collected after day 8 of illness from some patients whose initial serology was inconclusive to demonstrate specific IgG seroconversion

AM: Aseptic meningitis

E/ME: Encephalitis or meningo-encephalitis

IgM_{CSF}: IgM anti-WNV in cerebrospinal fluid

IgM_S: IgM anti-WNV in serum

IgG_S: IgG anti-WNV in serum

PCR_{CSF}: Real-time RT-PCR of WNV in cerebrospinal fluid

PCR_U: Real-time RT-PCR of WNV in urine

PRNT: Plaque Reduction Neutralization Test with at least 90% reduction at the 1:20 serum dilution

Table S4. Model comparison criteria and diagnostic checks for serocatalytic models of West Nile virus in Algeria, 2017-2018. For PSIS-LOO, lower values indicate better fit. Warnings about divergent transitions indicate that the model may be mis-specified. Bold indicates the best-fitting model for each province.

**Province**	**Region**	**Model**	**Number of parameters**	**R-hats below 1.01**	**Good mixing**	**PSIS-LOO***	**Difference in expected log pointwise predictive density (ELPD)**	**Standard error of expected log pointwise predictive density (ELPD)**	**Warnings about divergent transitions**
Oran	North	Recent outbreak	1	Yes	Yes	204.2	-6.1	2.8	No
		Constant	1	Yes	Yes	196.8	-2.4	2.9	78 divergent transitions after warmup
		Constant with seroreversion	2	Yes	Yes	195.7	-1.9	2.2	No
		**One outbreak**	**2**	**Yes**	**Yes**	**191.9 (8% of Pareto k diagnostic values are still too high with moment matching)**	**0**	**0**	**68 divergent transitions after warmup**
		Constant with outbreak	3	No, highest is 1.018	Yes but outbreak time is skewed	198.3 (5% of Pareto k diagnostic values are still too high with moment matching)	-3.2	3.4	121 divergent transitions after warmup
Tizi Ouzou	North	**Recent outbreak**	**1**	**Yes**	**Yes**	**82.8**	**0**	**0**	**No**

Province	Region	Model	Number of parameters	R-hats below 1.01	Good mixing	PSIS-LOO*	Difference in expected log pointwise predictive density (ELPD)	Standard error of expected log pointwise predictive density (ELPD)	Warnings about divergent transitions
		Constant	1	Yes	Yes	100.0	-8.6	4.9	62 divergent transitions after warmup
		Constant with seroreversion	2	Yes	Yes	89.2	-3.2	3.3	No
		One outbreak	2	Yes	Yes	88.4	-2.8	2.1	470 divergent transitions after warmup
		Constant with outbreak	3	Yes	Yes	92.7 (1% of Pareto k diagnostic values are too high)	-5.0	2.5	189 divergent transitions after warmup
Jijel	North	**Recent outbreak**	**1**	**Yes**	**Yes**	**197.5**	**-0.3**	**0.7**	**No**
		Constant	1	Yes	Yes	208.3	-5.7	4.4	81 divergent transitions after warmup
		Constant with seroreversion	2	Yes	Yes	196.9	0	0	No
		One outbreak	2	Yes	Yes	198.0	-0.6	1.8	112 divergent transitions after warmup
		Constant with outbreak	3	Yes	Yes	200.4	-1.7	2.5	187 divergent transitions after warmup
Biskra	South	Recent outbreak	1	Yes	Yes	343.3	-28.7	7.2	No

Province	Region	Model	Number of parameters	R-hats below 1.01	Good mixing	PSIS-LOO*	Difference in expected log pointwise predictive density (ELPD)	Standard error of expected log pointwise predictive density (ELPD)	Warnings about divergent transitions
		**Constant**	**1**	**Yes**	**Yes**	**287.4**	**-0.7**	**1.3**	**70 divergent transitions after warmup**
		Constant with seroreversion	2	Yes	Yes	294.2	-4.1	2.6	No
		One outbreak	2	Yes	Yes	346.5 (<1% of Pareto k diagnostic values are too high)	-30.3	7.2	68 divergent transitions after warmup
		Constant with outbreak	3	Yes	Yes	285.9 (All Pareto k estimates are good with moment matching)	0	0	59 divergent transitions after warmup
Timimoun	South	Recent outbreak	1	Yes	Yes	164.3	-10.3	4.3	No
		**Constant**	**1**	**Yes**	**Yes**	**144.7**	**-0.6**	**0.6**	**87 divergent transitions after warmup**
		Constant with seroreversion	2	Yes	Yes	147.5	-1.9	2.2	No
		One outbreak	2	Yes	Yes	172.4 (<1% of Pareto k diagnostic values are too high)	-14.4	4.5	66 divergent transitions after warmup

Province	Region	Model	Number of parameters	R-hats below 1.01	Good mixing	PSIS-LOO*	Difference in expected log pointwise predictive density (ELPD)	Standard error of expected log pointwise predictive density (ELPD)	Warnings about divergent transitions
		Constant with outbreak	3	Yes	Yes	143.6 (10% of Pareto k diagnostic values are still too high with moment matching)	0	0	81 divergent transitions after warmup

*Unless otherwise indicated, all Pareto k values were classified as good without the use of moment matching.
