# Supplementary material for: Patterns of emergence and circulation of West Nile Virus in Algeria
Source: Nat Commun. 2026 Jul 6;17:8370. doi: 10.1038/s41467-026-74856-6 (PMC13473090; doi:10.1038/s41467-026-74856-6)
Supplement: Supplementary file 2 [file 41467_2026_74856_MOESM2_ESM.pdf]

nature portfolio

Peer Review File

Patterns of Emergence and Circulation of West Nile Virus in Algeria

Corresponding Author: Professor Aissam Hachid

This file contains all reviewer reports in order by version, followed by all author rebuttals in order by version.

Version 0:

Reviewer comments:

Reviewer #1

(Remarks to the Author)
This is an interesting study aimed at improving the knowledge on the epidemiological pattern of WNV infections, including WNND, in Algeria by conducting a cross-sectional seroprevalence survey in humans and by fitting a serocatalytic models to the data collected. Results show an average sroprevalence 16.7% and identified the main risk factors. Also the patterns of spread in provinces in the south compared to those in the north are analyzed and discussed.
Overall, this paper contain very relevant and updated epidemiological information based on an adequate methodology and survey design. However, it would benefit from a major revision, especially of the discussion that should be re-organized taking into account the various factors considered, from the ecological to the demographic ones. Also the provisioning of a description of the main geographical and ecological features, including the major changes occurred during the last decades which characterize Algeria and the study sites, would improve the quality and relevance of the study
Some additional comments:
Line 50. Add other mosquitoes genera and species that are reported to transmit WNV
Line 53. Add other vertebrate species that are listed as competent reservoir for WNV
Line 57. Complete the list of risk factors, including the gender dimension
Line 70. According to ECDC the worst WNV epidemic in EU was recored in 2022 not in 2018. This statement need to be revised and reference need to be udated
Line 71. Climate change is only one of the various drivers affecting WNV ecology and epidemiology. This statement need to be revised and expanded and references updated

Reviewer #2

(Remarks to the Author)
The paper is exploring the Patterns of Emergence and Circulation of West Nile Virus in Algeria.
The data give new insights on West Nile circulation in the north versus the south of Algeria and the use of original serocatalytic models allow the authors to reconstruct the historical circulation of West Nile virus in the country.
The study is of high significance to the field of epidemiology on infectious diseases and the work presented support the claims and conclusions.
However, the methodology needs more clarification.
Below are some concerns that need to be addressed before publication:

L96-100 : this sentence « we conducted a cross-sectional seroprevalence study of WNV infection and analyzed data on WNND 98 cases » is confusing. The authors should clarify that they will perform in the 5 districts, a seroprevalence study as well as WNVD acute case detection in hospitals among patients with suspected viral neuroinvasive diseases. This will make the rest of the manuscript clearer

Leftover serum samples were collected from public and private laboratories from patients either seeking routine checkup analyses or displaying different pathologies with no bias toward a particular set of symptoms. The authors should specify the different pathologies
Sera from subjects with ongoing infectious diseases were discarded : does this mean that all samples patients with fever

were discarded ?

WNND case confirmation
L142 : is aseptic meningitis or ME/E mandatory in the case definition used for WNVD case detection ? Specify the case definition used for WND case detection in hospitals
Testing algorithm needs more clarification !
L150 : Additional follow-up serum samples were collected from some patients to demonstrate specific IgG seroconversion. The results of the follow up samples were not presented in the result section !
L172-180 : Are sera and CSF samples tested by RT-PCR ? or only urine samples were tested by RT-PCR and other samples by ELISA and PRNT ?
For the 206 samples positive by PRNT, what was the titer range ?
L264 : the 31 WNND confirmed cases were positive by RT-PCR or only by IgM and PRNT ? Are the IgM/IgG positive samples considered in the serocatalytic models or only IgG samples detected in the seroprevalence study were considered ?
L387 : Sindbis is an alphavirus and not a flavivirus ! This vorus virus should not give cross reactions with West Nile. However, Usutu, belonging to the same serocomplex as West Nile can give cross reaction even with PRNT !
Then to rule out cross reactions with Usutu, differential ELISA and PRNT should also be tested. If the tests are not available for Usutu, this should be discussed as probable limitation

Reviewer #3

(Remarks to the Author)
The authors utilized serological data from five provinces in Algeria to describe epidemiological patterns in and estimate the historical pattern of West Nile virus (WNV) transmission. This clearly written and justified manuscript is a valuable contribution to understanding the current distribution and patterns of WNV transmission. The descriptive analysis highlighted geographic variation in seroprevalence (higher seroprevalence in the south vs. the north) along with factors associated with increased exposure to WNV (living in a mosquito-infested area, engaging in outdoor activity). The serocatalytic models provided evidence of continual transmission in the southern provinces while the northern provinces likely have only experienced single, isolated outbreaks. These findings could be used to guide further surveillance and target prevention measures to communities of high risk.
I have a few, minor comments and questions for the authors to consider.
The authors characterize WNV as an emerging arbovirus (page 1, line 49). Given its endemicity in many areas around the globe, “emerging” would not be the most appropriate term. Consider rephrase or qualifying this statement.
I have a few comments related to limitations of fitting the serocatalytic models. Given the large number of divergent transitions in all “best” models in the three northern provinces, further explanation and discussion on the potential impacts of these on the results is warranted. Also, were there additional steps that could further reduce divergent transitions beyond increasing the adapt_delta parameter and thinning posterior samples?
Figure 2. Consider reordering the panels to correspond to the relative geographic location of these provinces in Algeria. This would provide some visual continuity between the map presented in Figure 1 and the serological data presented in Figure 2.

Version 1:

Reviewer comments:

Reviewer #2

(Remarks to the Author)
The proposed answers to the various questions are very satisfactory. The methodology is much clearer. I have no additional comments or questions.

Reviewer #3

(Remarks to the Author)
Thank you to the authors for their revisions. The clarity of the manuscript has increased. I appreciate the multi-model comparison the authors undertook to assess the plausibility of WNV transmission dynamics. I have a few comments in regards to providing clarity and details.
Lines 210-216. Was the multivariate model fitted to the same observations across all steps of the backwards elimination process? Was the final multivariate model refitted using observations with non-missingness for only the selected covariates? For example, outdoor exposure would have been initially included in the multivariate model (P-value < 0.2 in univariate logistic model), but this covariate had a high amount of missingness across observations (63.8% missing). When this covariate was removed due to non-significance in the process (it is not listed in the final model), additional observations could have been available for inclusion as they were not missing for the subset of variables remaining in the model. However, including these in future backward elimination steps means the models were fitted with different sample sizes and not comparable. I imagine that a single set of observations were used to select the final multivariate model such that model comparisons were valid throughout the process. I also assume that the presented multivariate model was refitted to with observations with non-missing values for the final considered covariates to produce the adjusted odds ratios. Please clarify if

these things did happen, and if not, why.
Lines 238. Perhaps a clearer name for model 3 would be “constant with outbreak” to align with the naming convention for model 2 (“constant with seroreversion”) and clarify that this model was not assuming a single, constant outbreak (i.e., differentiate from model 1).
Line 258. I suggest the authors compute and report differences in the estimated log point densities (ELPD) as part of the model selection process. The magnitude of the difference in ELPD and standard error of the difference provides an additional level of support for models being different or similar (see discussion and references: https://mc-stan.org/loo/articles/online-only/faq.html#elpd_interpretation).
Line 338. I recommend noting (again) the year of data collection in these provinces so readers don’t miss these in the Methods.
Lines 347-349. Did both alternative prior distributions result in divergent transitions and algorithm failure or did each result in a different “failure”? If different, please indicate which prior resulted in what outcome. On a similar note, was it just the models for these two provinces that resulted in estimates with negative outbreak timings? Why did the authors use the default prior for outbreak timing if it included nonsensical negative values?
Lines 388-394. Even though the outbreak models fitted worse than the recent outbreak models for both Jijel and Tizi Ouzou, did the fitted peak year of outbreak align with these 2012 reports of WNV infection? Curious if the models provided support for earlier transmission or if the heterogeneity in networks mentioned could have been a factor in local WNV transmission dynamics.
Lines 413-422. This paragraph provides a summary of reported seroprevalence from a similar geographic area. However, further conclusions and synthesis is warranted here.
Line 439. Another limitation or area of discussion would be the various fits of the models and thus their plausibility, especially given the indications of lack of fit (e.g., high Pareto k, divergent transitions). I’m not saying that the models or conclusions drawn are wrong, but that the indications of lack of fit indicate further data and/or investigation is warranted.
Figure 2. Having a different color per panel is not necessary since the paneling itself already illustrates each panel presents the results for a different province. However, color could be used to provide visual continuity throughout the manuscript. For example, the same colors could be used between Fig 2 and Fig 3A for denoting provinces. In a similar vein, I suggest harmonizing the colors used for denoting ME/E and MLC (Fig 3B and Fig S2).
Table 3. The attack rates and annual probability of infection are presented with different numbers of significant digits such that it appears to be different levels of precision. Please harmonize the number of digits presented.
Table S3. As there are no warnings about low effective sample sizes presented in the table, reference to this warning could be removed in the table header and caption.
Additionally, an explanation of the impact of the listed Pareto k warning should be included to enable readers to understand the implications of such warning. This warning is often associated with model misspecification. In Lines 260-263, it appears that moment matching was used to correct for high Pareto k values. Does the warning about high values indicate problematic values even after using moment matching or were these warnings that were corrected after using moment matching?

Version 2:

Reviewer comments:

Reviewer #3

(Remarks to the Author)
Thank you to the authors for their diligent revisions and patience in this review process. The clarity of the manuscript has increased and represents a large amount of multi-disciplinary work. All my comments have been addressed satisfactorily.

Open Access This Peer Review File is licensed under a Creative Commons Attribution 4.0 International License, which permits use, sharing, adaptation, distribution and reproduction in any medium or format, as long as you give appropriate credit to the original author(s) and the source, provide a link to the Creative Commons license, and indicate if changes were made.
In cases where reviewers are anonymous, credit should be given to 'Anonymous Referee' and the source.
The images or other third party material in this Peer Review File are included in the article's Creative Commons license, unless indicated otherwise in a credit line to the material. If material is not included in the article's Creative Commons license and your intended use is not permitted by statutory regulation or exceeds the permitted use, you will need to obtain permission directly from the copyright holder.
To view a copy of this license, visit https://creativecommons.org/licenses/by/4.0/

REVIEWER COMMENTS

Reviewer #1 (Remarks to the Author):

This is an interesting study aimed at improving the knowledge on the epidemiological pattern of WNV infections, including WNND, in Algeria by conducting a cross-sectional seroprevalence survey in humans and by fitting a serocatalytic models to the data collected. Results show an average seroprevalence 16.7% and identified the main risk factors. Also the patterns of spread in provinces in the south compared to those in the north are analyzed and discussed.
Overall, this paper contain very relevant and updated epidemiological information based on an adequate methodology and survey design. However, it would benefit from a major revision, especially of the discussion that should be re-organized taking into account the various factors considered, from the ecological to the demographic ones. Also the provisioning of a description of the main geographical and ecological features, including the major changes occurred during the last decades which characterize Algeria and the study sites, would improve the quality and relevance of the study.

We thank the reviewer for the valuable suggestion. We have restructured the discussion to improve clarity. The discussion is now structured into 8 paragraphs:

Paragraph 1: Summary of the study's objectives.
Paragraph 2: Discussion of the major findings of the study that WNV has been endemic in southern Algeria for decades but has only recently emerged in the north.
Paragraph 3: Discussion of the consistency of our modeling findings with surveillance data on clinical cases of WNND, enhancing understanding of the virus's transmission.
Paragraph 4: Discussion on the impact of climate and socioecological changes on the WNV patterns in Algeria.
Paragraph 5: Comparison of our epidemiological results with those reported in neighboring and Mediterranean countries.
Paragraph 6: Identification of factors associated with WNV seropositivity, including age, geographic region, and rural residence.
Paragraph 7: Overview of the different limitations and strengths of our study
Paragraph 8: Conclusions from our work.

We also added a detailed description of how climate and socioecological changes have impacted the spread of WNV in Algeria. For example, in lines 401-412, we have written:

"Profound demographic and environmental transformations, driven by demographic growth and socio-economic changes, have occurred in Algeria's cities and rural areas in recent decades. In the north, dense human settlements, irrigation systems, wetlands, and rapid urban development have created favorable conditions for mosquito breeding and development. Algeria has also experienced major climatic change, including rising temperatures and decreased rainfall, especially in the north, which have likely affected vector ecology and transmission of viruses. These factors combined have led to the

spread of WNV into new areas. Indeed, previous studies conducted in Europe, especially in the Mediterranean region, have shown an association between climate change and the rise in human WNV cases, which could be attributed to the expansion of *Culex* species and an increase in their ability to transmit the virus."

Some additional comments:
Line 50. Add other mosquitoes genera and species that are reported to transmit WNV

Thanks for the suggestion. Here is the updated text from lines 50-53:

"It [WNV] is primarily transmitted to humans through the bite of infected mosquitoes, predominantly by *Culex* species such as *Culex pipiens* complex and *Culex perexiguus*. Other mosquito species, including *Aedes albopictus* and *Aedes rusticus*, have been reported to transmit WNV."

Line 53. Add other vertebrate species that are listed as competent reservoir for WNV

Birds are the only well-documented competent animal reservoir for WNV. While other non-avian vertebrae species, such as mammals, humans, and horses, are susceptible, they do not develop high enough viremia to transmit the disease and are therefore considered 'dead end' hosts. However, not all bird species are considered competent reservoirs.
Sources: https://doi.org/10.1080/14787210.2020.1713751
doi: 10.1080/22221751.2024.2437244
https://www.cdc.gov/west-nile-virus/php/transmission/index.html
https://doi.org/10.1371/journal.pone.0292187
https://doi.org/10.1016/B978-0-12-374271-1.00045-9

This statement was modified in the main text (lines 56-58).

"WNV has an enzootic transmission cycle involving migratory or resident birds, which are the natural reservoirs. Most of these birds act as amplifying hosts. Horses, humans, and other mammals are considered dead-end hosts."

Line 57. Complete the list of risk factors, including the gender dimension

Thanks, we have expanded the list of risk factors in lines 62-64:

"Risk factors for severe forms of WNV include older age, underlying medical conditions, immune suppression, male sex, and exposure via blood transfusion or organ transplantation."

Line 70. According to ECDC the worst WNV epidemic in EU was recorded in 2022 not in 2018. This statement need to be revised and reference need to be updated

The statement in the text is correct. According to the ECDC, the largest epidemic in Europe occurred in 2018, resulting in 2,083 WNV cases and 180 deaths in humans (https://www.ecdc.europa.eu/en/news-events/epidemiological-update-west-nile-virus-transmission-season-europe-2018). In 2022, there were 1,340 cases, including 104 deaths, among humans (https://www.ecdc.europa.eu/en/news-events/epidemiological-update-west-nile-virus-transmission-season-europe-2022#:~:text=As%20of%2030%20June%202023,the%20peak%20epidemic%20year%202018).

Line 71. Climate change is only one of the various drivers affecting WNV ecology and epidemiology. This statement need to be revised and expanded and references updated

Thank you for highlighting this important point. We have revised the text to provide more details on the various factors influencing WNV epidemiology and ecology, and updated the references accordingly. See lines 76-79:

“Viral evolution, ecological changes influencing the abundance and distribution of WNV vectors and hosts (e.g., land use and urbanization), and climate change, through increasing temperatures and fluctuating rainfall, may contribute to the spread and establishment of WNV in different parts of the world.”

Sources:

Camp, J. V. & Nowotny, N. The knowns and unknowns of West Nile virus in Europe: what did we learn from the 2018 outbreak? *Expert Review of Anti-infective Therapy* **18**, 145–154 (2020).

Chancey, C., Grinev, A., Volkova, E. & Rios, M. The Global Ecology and Epidemiology of West Nile Virus. BioMed Research International 2015, 1–20 (2015).

World Health Organization. West Nile virus. https://www.who.int/news-room/fact-sheets/detail/west-nile-virus.

Paz, S. Climate change impacts on West Nile virus transmission in a global context. *Phil. Trans. R. Soc. B* **370**, 20130561 (2015).

Mencattelli, G. *et al.* Spatial and temporal dynamics of West Nile virus between Africa and Europe. *Nat Commun* **14**, 6440 (2023).

Reviewer #2 (Remarks to the Author):

The paper is exploring the Patterns of Emergence and Circulation of West Nile Virus in Algeria.

The data give new insights on West Nile circulation in the north versus the south of Algeria and the use of original serocatalytic models allow the authors to reconstruct the historical circulation of West Nile virus in the country.
The study is of high significance to the field of epidemiology on infectious diseases and the work presented support the claims and conclusions.
However, the methodology needs more clarification.
Below are some concerns that need to be addressed before publication:

L96-100 : this sentence « we conducted a cross-sectional seroprevalence study of WNV infection and analyzed data on WNND 98 cases » is confusing. The authors should clarify that they will perform in the 5 districts, a seroprevalence study as well as WNVD acute case detection in hospitals among patients with suspected viral neuroinvasive diseases.
This will make the rest of the manuscript clearer

> Thanks for pointing out this issue with clarity. We have re-phrased this sentence in lines 106-110 as follows:
>
> "Here, we sought to gain further insight into the epidemiology of WNV in Algeria. Across five provinces with suitable environmental conditions for WNV transmission and/or previous reports of human cases of WNV infections, we conducted a seroprevalence study as well as WNV disease acute case detection in hospitals among patients with suspected viral neuroinvasive disease."

Leftover serum samples were collected from public and private laboratories from patients either seeking routine checkup analyses or displaying different pathologies with no bias toward a particular set of symptoms. The authors should specify the different pathologies Sera from subjects with ongoing infectious diseases were discarded :
does this mean that all samples patients with fever were discarded ?

> We thank the reviewer for this important comment. This information has now been specified in the Methods section in lines 146-149:
>
> "Sera from subjects with immunological disorders or ongoing infectious disease (which required immunological, microbiological, or virological investigations) or those presenting with fever were discarded to reduce bias."

WNND case confirmation
L142 : is aseptic meningitis or ME/E mandatory in the case definition used for WNVD case detection ? Specify the case definition used for WND case detection in hospitals. Testing algorithm needs more clarification !

> We appreciate your observation. According to the Algerian Ministry of Health WNV surveillance system, the presence of aseptic meningitis or ME/E is mandatory in the case definition for WNND. To avoid ambiguity, we have added a dedicated paragraph in

the Methods section that clearly states the official case definition used in our study (lines 157-161):

"In line with the Algerian Ministry of Health surveillance framework, a suspected case of WNND was defined as a patient exhibiting neurological manifestations (e.g., aseptic meningitis or meningoencephalitis), along with fever (≥38.5°C) and cerebrospinal fluid (CSF) showing a lymphocytic predominance (>50%) and normal glycorrhachia, where a viral etiology could not be excluded."

We also added a paragraph in methods section (below, lines 170-175) and included the testing algorithm for WNND in Figure S1 with further details in supplementary methods.

"A confirmed case of WNND met at least one of the following laboratory criteria: (i) detection of WNV-specific IgM antibody in CSF by ELISA, (ii) detection of WNV RNA in urine and/or CSF by PCR, or (iii) detection of IgM and IgG antibodies in serum by ELISA confirmed by detection of WNV neutralizing antibodies using the plaque reduction neutralization test (PRNT). Further details about the diagnostic testing algorithm can be found in Supplementary methods and Figure S1."

L150 : Additional follow-up serum samples were collected from some patients to demonstrate specific IgG seroconversion. The results of the follow up samples were not presented in the result section !

We appreciate your comment. Follow-up sera were collected from 4 patients who had isolated anti-WNV IgM detected in their acute serum at the time of hospitalization.

The results of the follow-up samples are presented in Table S2.

L172-180 : Are sera and CSF samples tested by RT-PCR ? or only urine samples were tested by RT-PCR and other samples by ELISA and PRNT ?

We appreciate your valuable comments. Acute WNV infection was confirmed by RT-PCR on urine and/or CSF samples when available. For cases where these specimens were missing or RT-PCR results were negative, confirmation was conducted by PRNT. We have added the testing algorithm in Figure S1 and more details in Supplementary methods (pages 3-4):

"When a patient is suspected of having WNND, the first step is to test serum for West Nile virus-specific IgM using ELISA (Figure S1). If this test is positive, further confirmation tests, including those for IgG in serum, IgM in cerebrospinal fluid (CSF), and/or PCR (in urine and/or CSF), as well as PRNT90 in serum, may be required depending on which samples are available. If IgM is not initially detected in serum, a

follow-up ELISA is required on serum collected after at least day 8 of illness onset to assess seroconversion. A WNND case is confirmed if at least one of the following is true: (i) WNV-specific IgM antibodies are detected in CSF, (ii) WNV RNA is detected in urine and/or CSF by PCR, or (iii) IgM and IgG antibodies are detected in serum by ELISA followed by detection of WNV neutralizing antibodies by PRNT90. If none of the criteria are met, an alternative diagnosis should be explored. Note that when CSF volume is limited, PCR is prioritized over IgM ELISA; otherwise, PCR is performed regardless of IgM CSF results for research purposes (e.g., strain genotyping)."

Also see Table S2 for details about the virological findings of the 31 WNND cases.

For the 206 samples positive by PRNT, what was the titer range ?

As described in the Methods section of the main text, we performed a qualitative PRNT90 using a single serum dilution (1:20), tested in duplicate to allow screening of a large number of samples, with positivity defined as ≥ 90% plaque reduction at this dilution.

L264 : the 31 WNND confirmed cases were positive by RT-PCR or only by IgM and PRNT ? Are the IgM/IgG positive samples considered in the serocatalytic models or only IgG samples detected in the seroprevalence study were considered ?

Of the 31 WNND cases, 8 were confirmed by PCR (in urine and/or CSF), while the remaining cases were confirmed serologically (IgM, IgG and PRNT). To improve clarity, we have added the following text to the results section (lines 304-309):

"As summarized in Table S2, most patients tested positive for IgM in serum and/or CSF. These results were often accompanied by a positive IgG test. In four patients with inconclusive serology, follow-up ELISA testing revealed seroconversion. The diagnosis of WNND was further supported by PCR detection of WNV RNA in urine or CSF in 8 cases, and by the identification of neutralizing antibodies through PRNT90 screening in 23 patients."

We have also added the testing algorithm in Figure S1 and Table S2 which describes the virological findings of the 31 WNND cases.

Only samples from the seroprevalence study were considered in the serocatalytic models. We have clarified this point in the methods on lines 234-236:

"For each study location, we used the RSero package in R to fit five models representing different patterns of transmission to the seroprevalence data (not the WNND data)."

L387 : Sindbis is an alphavirus and not a flavivirus ! This virus should not give cross reactions with West Nile. However, Usutu, belonging to the same serocomplex as West Nile can give cross reaction even with PRNT !
Then to rule out cross reactions with Usutu, differential ELISA and PRNT should also be tested. If the tests are not available for Usutu, this should be discussed as probable limitation

> Thanks for catching this! We have removed Sindbis from our list of flaviviruses and added the following additional information about Usutu virus (lines 449-453):
>
> "Since specific tests for Usutu virus, which belongs to the same serocomplex as WNV and can cross-react even with PRNT, are not available in the country, we did not perform differential tests. However, recent studies indicate that Usutu virus circulation in Algeria is limited and is unlikely to have substantially influenced our results."

Reviewer #3 (Remarks to the Author):

The authors utilized serological data from five provinces in Algeria to describe epidemiological patterns in and estimate the historical pattern of West Nile virus (WNV) transmission. This clearly written and justified manuscript is a valuable contribution to understanding the current distribution and patterns of WNV transmission. The descriptive analysis highlighted geographic variation in seroprevalence (higher seroprevalence in the south vs. the north) along with factors associated with increased exposure to WNV (living in a mosquito-infested area, engaging in outdoor activity). The serocatalytic models provided evidence of continual transmission in the southern provinces while the northern provinces likely have only experienced single, isolated outbreaks. These findings could be used to guide further surveillance and target prevention measures to communities of high risk.
I have a few, minor comments and questions for the authors to consider.
The authors characterize WNV as an emerging arbovirus (page 1, line 49).
Given its endemicity in many areas around the globe, "emerging"
would not be the most appropriate term. Consider rephrase or qualifying this statement.

> We have removed the word "emerging" from the abstract and introduction.

I have a few comments related to limitations of fitting the serocatalytic models. Given the large number of divergent transitions in all "best" models in the three northern provinces, further explanation and discussion on the potential impacts of these on the results is warranted. Also, were there additional steps that could further reduce divergent transitions beyond increasing the adapt_delta parameter and thinning posterior samples?

> We have re-run all the models with the new version of the Rsero package which substantially decreased the number of divergent transitions. The new version of the package uses a non-centered parameterization for the parameters when a normal or lognormal prior distribution is specified. We were able to run the models with fewer iterations, the default value for adapt_delta of 0.95, and no thinning. The new version

also includes additional model comparison criteria, including WAIC and PSIS-LOO, which we decided to use instead of DIC. PSIS-LOO is the preferred method for comparing Stan models (https://link.springer.com/article/10.1007/s11222-016-9696-4). Finally, we included a check for R-hat values in Table S3.

We also fitted a new model, called the “recent outbreak” model, which became the best-fitting model for two of the northern provinces.

Figure 2. Consider reordering the panels to correspond to the relative geographic location of these provinces in Algeria. This would provide some visual continuity between the map presented in Figure 1 and the serological data presented in Figure 2.

This is a great suggestion - we have re-ordered the panels as suggested.

Thanks again for the opportunity to revise the paper.

Sincerely,
Dr. Hachid et al.

REVIEWER COMMENTS

Reviewer #3 (Remarks to the Author):

Thank you to the authors for their revisions. The clarity of the manuscript has increased. I appreciate the multi-model comparison the authors undertook to assess the plausibility of WNV transmission dynamics. I have a few comments in regards to providing clarity and details.

Lines 210-216. Was the multivariate model fitted to the same observations across all steps of the backwards elimination process?

No, it was not, but we have re-run the model with the same number of observations across all steps of the backwards elimination process, and our results do not change. We have updated the methods as follows in lines 212-222:

"We also performed multivariable logistic regression using a stepwise down procedure. All variables with a p-value < 0.20 in the univariable analysis (except for "outdoor activity" which had over 60% of observations missing) were initially included in the multivariable logistic regression model. Variables with the highest p value were removed from the model one at a time until all remaining variables were statistically significant at the 0.05 level. The multivariable model was fitted to the same observations across all steps of the stepwise down procedure. The final multivariable model was refitted using non-missing observations for only the selected covariates. For each variable retained in the final model, adjusted odds ratios (AOR) were calculated along with their 95% CI. We used 2-tailed tests for all logistic regression models."

We have also added a footnote to Table 1 indicating the number of observations included in both the stepwise down procedure and the final, refitted model:

"In the multivariable analysis, 1,060 non-missing observations were included in all steps of the stepwise down procedure, while 1,224 non-missing observations were included in the final, refitted model."

Was the final multivariate model refitted using observations with non-missingness for only the selected covariates? For example, outdoor exposure would have been initially included in the multivariate model (P-value < 0.2 in univariate logistic model), but this covariate had a high amount of missingness across observations (63.8% missing). When this covariate was removed due to non-significance in the process (it is not listed in the final model), additional observations could have been available for inclusion as they were not missing for the subset of variables remaining in the model. However, including these in future backward elimination steps means the models were fitted with different sample sizes and not comparable. I imagine that a single set of observations were used to select the final multivariate model such that model comparisons were valid throughout the process. I also assume that the presented multivariate model was refitted to observations with non-missing values for the final considered covariates to produce the adjusted odds ratios. Please clarify if these things did happen, and if not, why.

Yes, the final model was refitted using observations with non-missingness for only the selected covariates. We have clarified this in the text and with a new footnote to Table 1 (see above).

Lines 238. Perhaps a clearer name for model 3 would be “constant with outbreak” to align with the naming convention for model 2 (“constant with seroreversion”) and clarify that this model was not assuming a single, constant outbreak (i.e., differentiate from model 1).

We have updated the name for model 3 to “constant with outbreak” as suggested. We also updated the description of this model as follows (lines 250-251):

“The constant with outbreak model has a constant FOI (like the constant model), but one or more outbreaks can still occur.”

Line 258. I suggest the authors compute and report differences in the estimated log point densities (ELPD) as part of the model selection process. The magnitude of the difference in ELPD and standard error of the difference provides an additional level of support for models being different or similar (see discussion and references: https://urldefense.com/v3/__https://mc-stan.org/loo/articles/online-only/faq.html__;!!JFdNOqOXpB6UZW0!vBqfA4aU_Q6R1QPgg51EpMN0_Iev0EjZbeT1kv2-sparggy_meLsIPMy3iMbKYT-zniemiLfXBtZlRSKblxubQXRmg$[13]#elpd_interpretation).

Done. See the updated Table S3 in the Appendix. We have also added the following to the methods in lines 264-268:

“We also computed the difference in expected log pointwise predictive density (ELPD) and the standard error of the ELPD to provide an additional level of support for models being similar or different. If the difference in ELPD is less than 4, the difference between models is considered small."

Line 338. I recommend noting (again) the year of data collection in these provinces so readers don’t miss these in the Methods.

Good idea. We have added some details to that sentence as follows:

“Outbreaks may have occurred in these locations around the time of the data collection (2017 for Jijel and 2018 for Tizi Ouzou).”

Lines 347-349. Did both alternative prior distributions result in divergent transitions and algorithm failure or did each result in a different “failure”? If different, please indicate which prior resulted in what outcome. On a similar note, was it just the models for these two provinces that resulted in estimates with negative outbreak timings? Why did the authors use the default prior for outbreak timing if it included nonsensical negative values?

No, each resulted in a different “failure.” We clarify this point in the appendix (page 4):

“For both provinces, using an exponential distribution fixed the problem of estimating outbreak times in the future but greatly increased the number of divergent transitions by an order of magnitude. Initialization of the Markov chain Monte Carlo algorithm failed for both provinces using an alternative normal distribution (Figure S4).”

Yes, it was just the models for these two provinces that resulted in estimates with negative outbreak timings.

By default, Rsero uses non-centered parameterization for the parameters when a normal or lognormal prior distribution is specified. Non-centered parameterization is used to improve model efficiency, namely to increase the effective sample size for the same number of iterations and to improve convergence (source https://mc-stan.org/docs/2_18/stan-users-guide/reparameterization-section.html). However, this parameterization allows negative values.

We have included some information on this in the appendix to clarify.

Lines 388-394. Even though the outbreak models fitted worse than the recent outbreak models for both Jijel and Tizi Ouzou, did the fitted peak year of outbreak align with these 2012 reports of WNV infection? Curious if the models provided support for earlier transmission or if the heterogeneity in networks mentioned could have been a factor in local WNV transmission dynamics.

No. Using the default prior distributions for outbreak timing, we obtained a fitted peak year of mean 2016 (95% CrI: 2013 - 2025) for Jijel and 2021 (95% CrI: 2017 - 2030) for Tizi Ouzou (the credible intervals include years after data collection, or “in the future”). Using the exponential distribution with rate 0.1, we obtained a fitted peak year of mean 2015 (95% CrI: 2013 - 2018) for Jijel and 2018 (95% CrI: 2017 - 2019) for Tizi Ouzou. As mentioned above, the MCMC chains failed to initialize when using the alternative normal distribution (T1=35, T2=10) for outbreak timing.

Lines 413-422. This paragraph provides a summary of reported seroprevalence from a similar geographic area. However, further conclusions and synthesis is warranted here.

Thanks for the suggestion. We have added further conclusions and synthesis to the end of this paragraph:

“Whether study participants lived in rural or urban locations was not always clear in these studies. Three studies (Libya, Morocco, and Spain) did not include children, and it was unclear whether children were included in the studies in France and Tunisia. The Greek study was the only nationally representative study with a large sample size, while the study in Morocco had the smallest sample size of less than 100 individuals across two locations. The seroprevalence estimate from Egypt is similar to that for Biskra in our study (24% vs 29.1%). One study (in

Libya) used ELISA alone to confirm WNV serostatus which can lead to higher rates of false positives compared to using ELISA followed by PRNT. Differences in seroprevalence of WNV across locations may also be due to differences in human behavior, environmental conditions, and vector control interventions which affect the rate of human exposure to infected mosquitoes."

Line 439. Another limitation or area of discussion would be the various fits of the models and thus their plausibility, especially given the indications of lack of fit (e.g., high Pareto k, divergent transitions). I'm not saying that the models or conclusions drawn are wrong, but that the indications of lack of fit indicate further data and/or investigation is warranted.

Thanks, we have added the following limitation to the discussion (lines 454-457):

"Also, the best-fitting models for three out of five provinces had some divergent transitions after warmup and one out of five models had 8% of Pareto k diagnostic values that were too high, even after moment matching. Further data and/or investigation may be warranted given these indications of lack of fit."

Figure 2. Having a different color per panel is not necessary since the paneling itself already illustrates each panel presents the results for a different province. However, color could be used to provide visual continuity throughout the manuscript. For example, the same colors could be used between Fig 2 and Fig 3A for denoting provinces. In a similar vein, I suggest harmonizing the colors used for denoting ME/E and MLC (Fig 3B and Fig S2).

Thanks for the suggestion. We have changed the colors in Figure 3 and Figure S2 to denote provinces and severe forms of WNV disease.

Table 3. The attack rates and annual probability of infection are presented with different numbers of significant digits such that it appears to be different levels of precision. Please harmonize the number of digits presented.

Done.

**Province**	**Year of sample collection**	**Model**	**Attack rate, mean % (95% CrI)**	**Estimated time of outbreak, mean year (95% CrI)***	**Annual probability of infection, mean % (95% CrI)**
Oran	2018	One outbreak	14 (11 - 18)	2010 (2008 - 2012)	-
Tizi Ouzou	2018	Recent outbreak	7.2 (3.8 - 11)	-	-
Jijel	2017	Recent outbreak	10 (7.1 - 14)	-	-

Timimoun	2018	Constant FOI	-	-	2.2 (1.6 - 2.8)
Biskra	2018	Constant FOI	-	-	1.3 (1.1 - 1.6)

Table S3. As there are no warnings about low effective sample sizes presented in the table, reference to this warning could be removed in the table header and caption.

We have removed the reference to low effective sample sizes from the table header and caption.

Additionally, an explanation of the impact of the listed Pareto k warning should be included to enable readers to understand the implications of such warning. This warning is often associated with model misspecification.

We have added the following to the methods (lines 273-274):

“Very high Pareto k values typically suggest the model is mis-specified or that there are outliers or mistakes in data processing.”

In Lines 260-263, it appears that moment matching was used to correct for high Pareto k values. Does the warning about high values indicate problematic values even after using moment matching or were these warnings that were corrected after using moment matching?

We only used moment matching if more than 1% of Pareto k values were classified as too high. When we used it, moment matching often decreased the percentage of Pareto k values that were too high but did not eliminate the problem.

We have added a footnote to Table S3:

“Unless otherwise indicated, all Pareto k values were classified as good without the use of moment matching.”

Thanks,
Dr. Hachid et al.
