# Supplementary material for: Patterns of emergence and circulation of West Nile Virus in Algeria
Source: Nat Commun. 2026 Jul 6;17:8370. doi: 10.1038/s41467-026-74856-6 (PMC13473090; doi:10.1038/s41467-026-74856-6)
Supplement: Supplementary file 3 [file 41467_2026_74856_MOESM3_ESM.pdf]

nature portfolio

Corresponding author(s): Aissam Hachid, Simon Cauchemez

Last updated by author(s): May 13, 2026



# Reporting Summary

Nature Portfolio wishes to improve the reproducibility of the work that we publish. This form provides structure for consistency and transparency in reporting. For further information on Nature Portfolio policies, see our Editorial Policies and the Editorial Policy Checklist.

## Statistics

For all statistical analyses, confirm that the following items are present in the figure legend, table legend, main text, or Methods section.

| n/a | Confirmed | |
|---|---|---|
| ☐ | ☒ | The exact sample size ($n$) for each experimental group/condition, given as a discrete number and unit of measurement |
| ☐ | ☒ | A statement on whether measurements were taken from distinct samples or whether the same sample was measured repeatedly |
| ☐ | ☒ | The statistical test(s) used AND whether they are one- or two-sided<br>*Only common tests should be described solely by name; describe more complex techniques in the Methods section.* |
| ☐ | ☒ | A description of all covariates tested |
| ☐ | ☒ | A description of any assumptions or corrections, such as tests of normality and adjustment for multiple comparisons |
| ☐ | ☒ | A full description of the statistical parameters including central tendency (e.g. means) or other basic estimates (e.g. regression coefficient) AND variation (e.g. standard deviation) or associated estimates of uncertainty (e.g. confidence intervals) |
| ☐ | ☒ | For null hypothesis testing, the test statistic (e.g. $F$, $t$, $r$) with confidence intervals, effect sizes, degrees of freedom and $P$ value noted<br>*Give P values as exact values whenever suitable.* |
| ☐ | ☒ | For Bayesian analysis, information on the choice of priors and Markov chain Monte Carlo settings |
| ☒ | ☐ | For hierarchical and complex designs, identification of the appropriate level for tests and full reporting of outcomes |
| ☒ | ☐ | Estimates of effect sizes (e.g. Cohen's $d$, Pearson's $r$), indicating how they were calculated |

*Our web collection on statistics for biologists contains articles on many of the points above.*

## Software and code

Policy information about availability of computer code

| | |
|---|---|
| Data collection | No software was used |
| Data analysis | We used R software (version 4.3.2) for data analysis; logistic regression was used for the descriptive analysis and the Rsero package was used for the serocatalytic models. Figure 1 (the map) was generated using the free and Open Source QGIS software version 3.40.9. Code to perform the analysis is provided on GitHub (https://github.com/kcharniga/wnv_algeria). |

For manuscripts utilizing custom algorithms or software that are central to the research but not yet described in published literature, software must be made available to editors and reviewers. We strongly encourage code deposition in a community repository (e.g. GitHub). See the Nature Portfolio guidelines for submitting code & software for further information.

## Data

Policy information about availability of data

All manuscripts must include a data availability statement. This statement should provide the following information, where applicable:

- Accession codes, unique identifiers, or web links for publicly available datasets
- A description of any restrictions on data availability
- For clinical datasets or third party data, please ensure that the statement adheres to our policy

The aggregated seroprevalence data, including sample year, province, PRNT90 result (positive or negative), and age in 10-year groups, generated in this study have been deposited in the Zenodo database under accession code 10.5281/zenodo.20116898 (https://doi.org/10.5281/zenodo.20116898)
and are available on GitHub (https://github.com/kcharniga/wnv_algeria). The raw seroprevalence data are protected and are not available due to data privacy laws.

Researchers interested in accessing the study data are encouraged to submit a formal request to either the corresponding author (AH, ahachid@pasteur.dz or hachidaissam1@gmail.com) or his laboratory (email: laboratoire.arbovirus@pasteur.dz, phone: +213 20 32 23 62). To uphold ethical standards and ensure appropriate data use, each request will undergo a case-by-case review and approval process. Additionally, as the data include information collected in Algeria, access is subject to Algerian data ownership regulations and may require approval from relevant Algerian authorities. Final approval is expected within two months, and if access is granted, the duration of data access will be granted on a case-by-case basis. The data generated in this study for Figure 3 are provided in the Source Data file. Data on WNND cases can be found in Table S3.

## Research involving human participants, their data, or biological material

Policy information about studies with human participants or human data. See also policy information about sex, gender (identity/presentation), and sexual orientation and race, ethnicity and racism.

| | |
|---|---|
| Reporting on sex and gender | We report information about sex in Table 1. Sex was assigned by the healthcare practitioner. Information about gender was not available for our study. |
| Reporting on race, ethnicity, or other socially relevant groupings | We did not report race, ethnicity, or other socially relevant groupings in our study. |
| Population characteristics | We collected and analyzed data on age, sex, residence, and potential risk factors, such as outdoor activity. |
| Recruitment | For the seroprevalence study, leftover serum samples were collected from public and private laboratories from patients either seeking routine checkup analyses or displaying different pathologies with no bias toward a particular set of symptoms. For the analysis of severe cases, samples from patients with suspected viral neuroinvasive diseases were included in a national diagnostic protocol as part of the Algerian Ministry of Health surveillance program of WNV infections and submitted for diagnosis to the National Reference Center for Arboviruses and Emerging Viruses. |
| Ethics oversight | Ethics committee of Blida 1 University's faculty of medicine in Blida, Algeria (01/CEEC/2025) |

Note that full information on the approval of the study protocol must also be provided in the manuscript.

# Field-specific reporting

Please select the one below that is the best fit for your research. If you are not sure, read the appropriate sections before making your selection.

☒ Life sciences ☐ Behavioural & social sciences ☐ Ecological, evolutionary & environmental sciences

For a reference copy of the document with all sections, see nature.com/documents/nr-reporting-summary-flat.pdf

# Life sciences study design

All studies must disclose on these points even when the disclosure is negative.

| | |
|---|---|
| Sample size | For the seroprevalence study, the required sample size was estimated at a minimum of 800 individuals, with an average prevalence of 15% based on seroprevalence data in North African and Mediterranean countries, precision of 2.5%, and a 95% confidence interval (CI). |
| Data exclusions | For each variable, samples with missing information were excluded from the analysis for that variable. |
| Replication | All samples with positive and borderline results with the IgG ELISA test were further analyzed by the plaque reduction neutralization test (PRNT) to detect specific neutralizing antibodies for WNV. |
| Randomization | Randomization was not relevant because we did an observational, cross-sectional study. There was no intervention. |
| Blinding | Blinding was not relevant for this study because we did not do an experiment. We did an observational study. |

# Reporting for specific materials, systems and methods

We require information from authors about some types of materials, experimental systems and methods used in many studies. Here, indicate whether each material, system or method listed is relevant to your study. If you are not sure if a list item applies to your research, read the appropriate section before selecting a response.

## Materials & experimental systems

| n/a | Involved in the study |
|---|---|
| ☐ | ☒ Antibodies |
| ☐ | ☒ Eukaryotic cell lines |
| ☒ | ☐ Palaeontology and archaeology |
| ☒ | ☐ Animals and other organisms |
| ☐ | ☒ Clinical data |
| ☒ | ☐ Dual use research of concern |
| ☒ | ☐ Plants |

## Methods

| n/a | Involved in the study |
|---|---|
| ☒ | ☐ ChIP-seq |
| ☒ | ☐ Flow cytometry |
| ☒ | ☐ MRI-based neuroimaging |

# Antibodies

| | |
|---|---|
| Antibodies used | EUROIMMUN Anti-West Nile Virus ELISA (IgG) Order N°: EI 2662-9601 G; EUROIMMUN Anti-West Nile Virus ELISA (IgM) Ordre (catalogue) N° : EI 2662-9601 G |
| Validation | M. Niedrig, K. Sonnenberg, K. Steinhagen, J.T. Paweska. Comparison of ELISA and immunoassays for measurement of IgG and IgM antibody to West Nile virus in human sera against virus neutralization. Journal of Virological Methods, Volume 139, Issue 1,2007, Pages 103-105, ISSN 0166-0934, https://doi.org/10.1016/j.jviromet.2006.09.009 |

# Eukaryotic cell lines

Policy information about cell lines and Sex and Gender in Research

| | |
|---|---|
| Cell line source(s) | Porcine kidney stable (PS) cell lines were obtained from the Institut Pasteur de Dakar. The sex of the donor animal is unknown. |
| Authentication | None of the cell lines used in this study were authenticated. |
| Mycoplasma contamination | Cells were tested for mycoplasma contamination using a qPCR-based assay and were confirmed to be negative prior to use. |
| Commonly misidentified lines (See ICLAC register) | PS cells are not listed among commonly misidentified cell lines in the International Cell Line Authentication Committee register. |

# Clinical data

Policy information about clinical studies

All manuscripts should comply with the ICMJE guidelines for publication of clinical research and a completed CONSORT checklist must be included with all submissions.

| | |
|---|---|
| Clinical trial registration | We did not perform a clinical trial |
| Study protocol | We did not perform a clinical trial, and the study protocol can be found in the methods section of our paper |
| Data collection | For the seroprevalence study, the study locations included five provinces in Algeria: Jijel, Timimoun, Biskra, Oran, and Tizi Ouzou. Samples were collected from public and private laboratories from 2017-2018. For severe cases, the investigation focused on hospitalized patients residing in any of the five study locations with clinical presentation of aseptic meningitis or ME/E between June 1st and October 31st each year from 2015 to 2019. |
| Outcomes | For the seroprevalence study, the primary outcome was seropositivity to WNV by ELISA followed by PRNT90; for severe cases of WNND, the outcome was laboratory confirmation via WNV antibodies detection and PCR testing. |

# Plants

| | |
|---|---|
| Seed stocks | NA |
| Novel plant genotypes | NA |
| Authentication | NA |
